# Aqueous-Phase Multicomponent Reaction Mechanism for the Synthesis of Pyrido[2,3‑*d*]pyrimidines: A Theoretical Perspective

**DOI:** 10.1021/acsomega.5c03056

**Published:** 2025-06-19

**Authors:** Virginia C. Rufino, Giovanni W. Amarante, Hélio F. Dos Santos

**Affiliations:** Department of Chemistry, 28113 Federal University of Juiz de Fora, Rua José Lourenço Kelmer, Campus Universitário São Pedro, Juiz de Fora, Minas Gerais 36036-900, Brazil

## Abstract

A theoretical investigation of the reaction among benzaldehyde, Meldrum’s acid, and 6-aminouracil in aqueous solution is presented in this study, incorporating the impact of temperature on both the thermodynamic and kinetic aspects of the reaction. Five free energy profiles are presented, concerning the Knoevenagel condensation, Michael addition, cyclization, propanone, and CO_2_ release. We have also performed a simple kinetic analysis and a detailed microkinetic analysis. According to our results, the carbon–carbon bond formation between benzaldehyde and Meldrum’s acid is the rate-determining step. The temperature increases the reaction rate; however, it is still insufficient for the formation of pyrido­[2,3-*d*]­pyrimidines beyond trace amounts, in good agreement with experimental results. This explanation provides a thorough understanding of the multicomponent reaction mechanism that leads to the formation of pyrido­[2,3-*d*]­pyrimidines, thereby opening new possibilities for further exploration and optimization of the catalyzed process.

## Graphical abstract

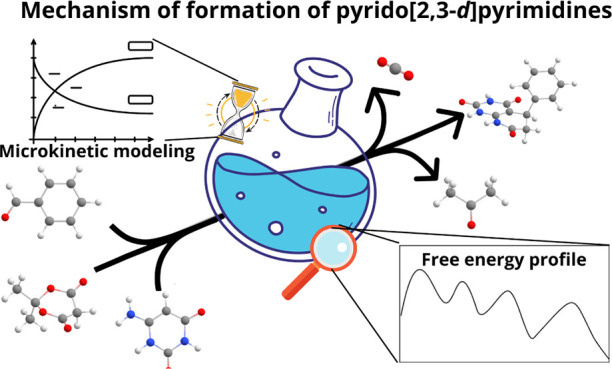

Pyrido­[2,3-*d*]­pyrimidine is one of the four possible isomers of pyridopyrimidines, and it plays an important role in biological activities.^1–3^ Scheme 1 shows different examples of pyrido­[2,3-*d*]­pyrimidine derivatives, where structures **1**–**5** exhibit antitumor activity,^4–8^ structure **6** exhibits antidepressant activity,^9^ structure **7** exhibits anticonvulsant activity,^9^ structure **8** exhibits α-glucosidase inhibition activity,^10^ and structure **9** exhibits antifungal activity.^11^ The interest in the biological activities of these structures motivates a synthetic focus on developing more efficient and sustainable methodologies.^12,13^

**Scheme 1:**
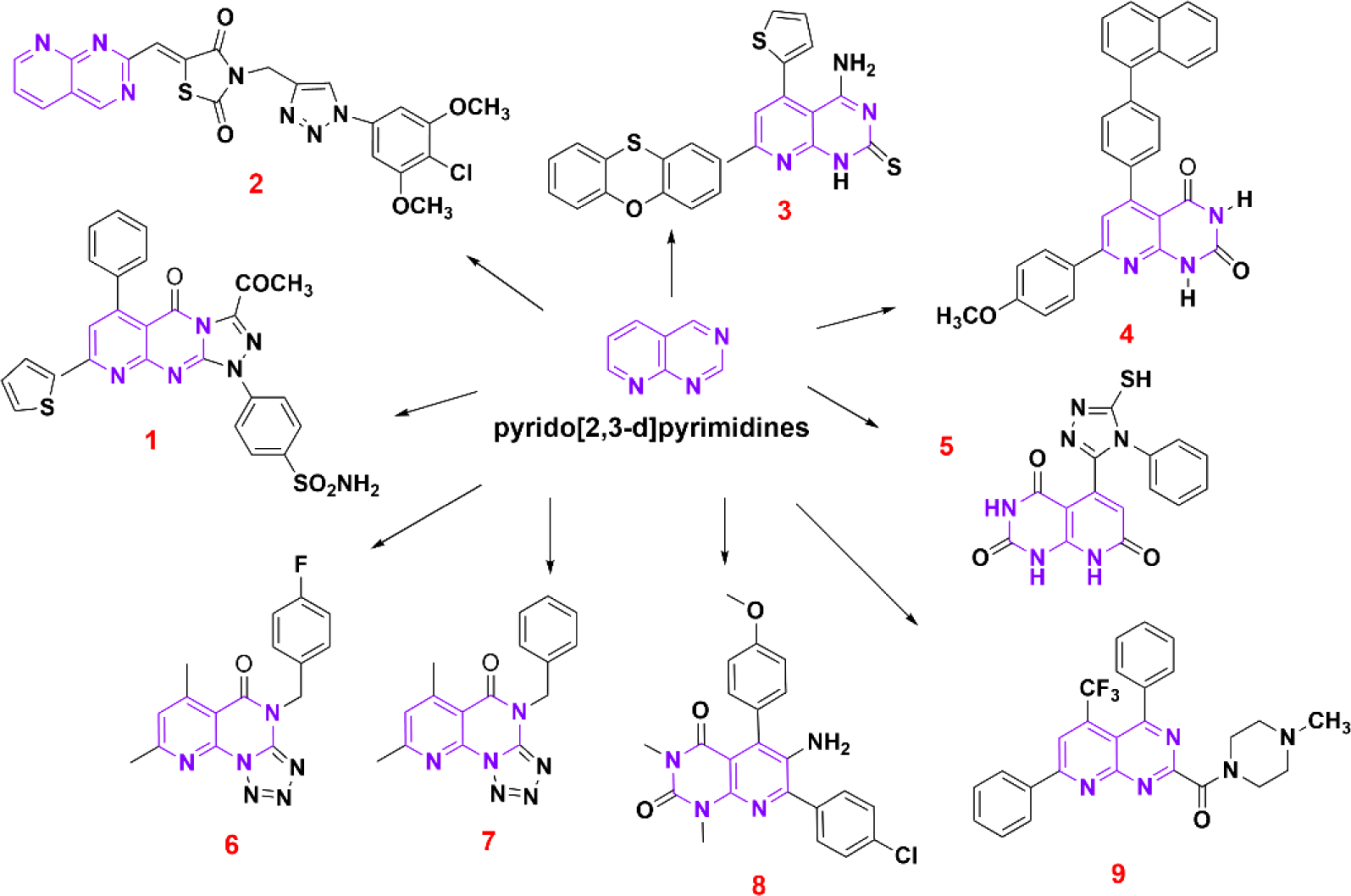
Structures with Biological Activity that Have a Core of Pyrido­[2,3-*d*]­pyrimidines

For a reaction to be considered sustainable, it must fulfill specific criteria, which start with the design of both the methodology and products. Some of these criteria include waste prevention, atom economy, use of innocuous solvents, shorter synthesis, and catalytic methodologies.^14,15^ According to the CHEM21 selection guide for classical solvents,^16^ the recommended solvents include water and certain types of alcohols. Water is ideally the better choice as a solvent for a reaction, presenting the best safety, health, and environmental scores. However, the use of water as a solvent does not necessarily make a reaction sustainable; the reaction also needs to meet the abovementioned criteria.

Multicomponent reactions (MCRs) are options that allow us to obtain the product in one-pot reactions with more than two starting compounds,^17^ avoiding unnecessary steps in synthesis, generating less waste, and achieving better atomic efficiency. Additionally, they can be conducted in aqueous solutions.^18,19^ The synthesis of pyrido­[2,3-*d*]­pyrimidines through the MCR methodology has been the subject of a series of experimental studies over the years (Scheme 2). In 2014, Mamaghani and coworkers made use of [γ-Fe_2_O_3_@-Hap-SO_3_H] as a nanocatalyst under solvent-free conditions to obtain pyrido­[2,3-*d*]­pyrimidines with up to 94% yield.^20^ In 2022, Bhat and Gupta conducted the reaction among Meldrum’s acid, benzaldehyde, and 6-amino-1,3-dimethyluracil, catalyzed by indium­(III) bromide (InBr_3_) under solvent-free conditions, and obtained the product with 95% yield in a reaction time of only 15 min.^21^ Despite the good results of these studies, the use of catalysts (as well as reagents) derived from renewable raw materials is preferable.^14^ In a 2020 study, Chate and coworkers proposed the use of β-cyclodextrin as a catalyst for the synthesis of pyrido­[2,3-*d*]­pyrimidines in aqueous solution under reflux conditions, and the desired product was obtained with up to 97% yield.^22^ This study represents a more sustainable approach for organic synthesis, with the potential for its methodology to be expanded to other MCRs, provided that the reaction mechanism and catalyst performance are initially understood.

**Scheme 2:**
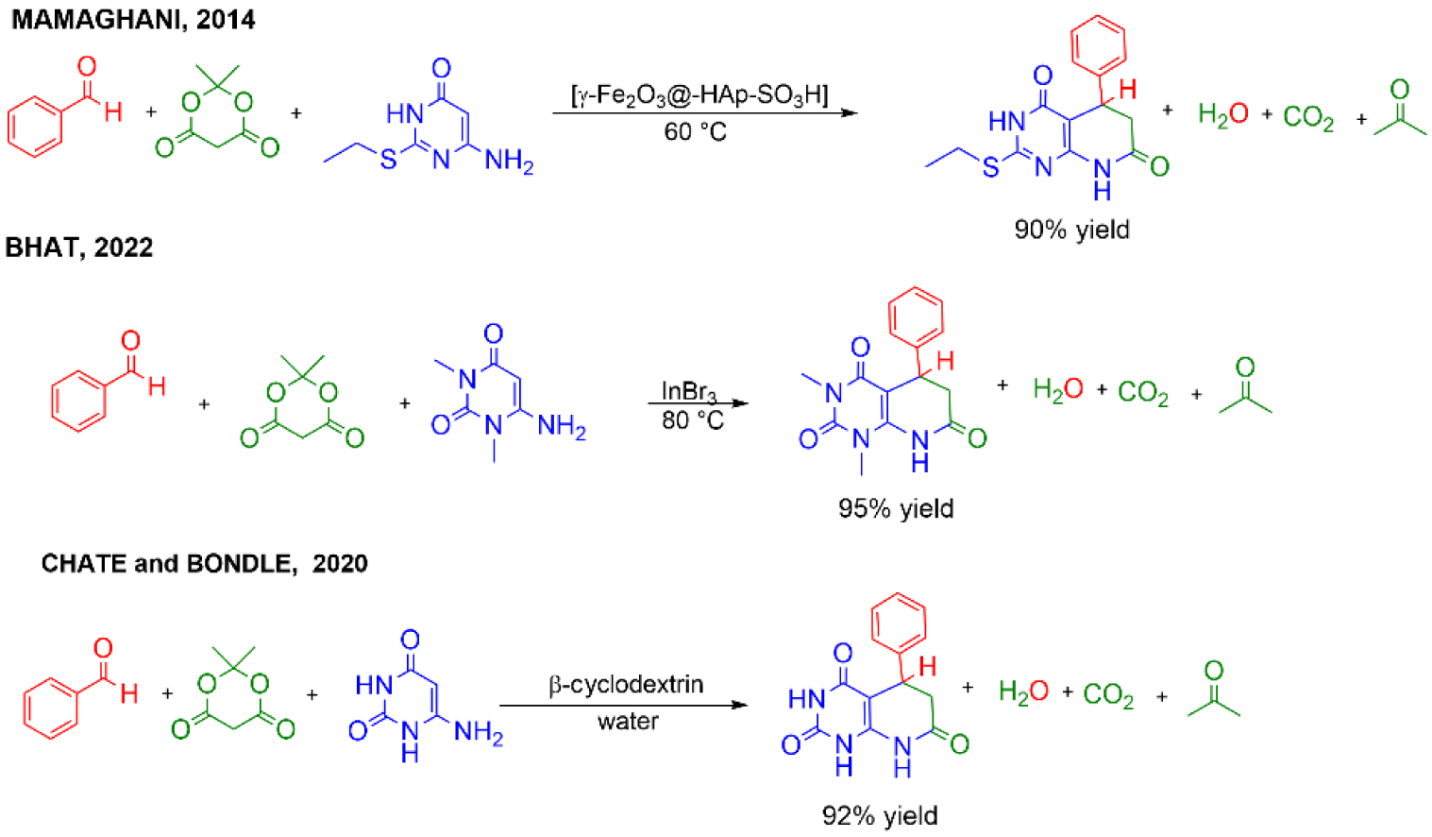
Reactions for the Formation of Pyrido­[2,3-*d*]­pyrimidines Conduced with Different Catalysts

The published papers in the literature only propose suggestions for reaction mechanisms,^21–23^ leaving a gap regarding the intermediates, transition states, and which step is the rate-determining step. Building on those proposals, we refined the mechanism presented in Scheme 3. The reaction unfolds through five mechanistic steps: Knoevenagel condensation, Michael addition, cyclization, propanone, CO_2_ release, and tautomerization, each comprising multiple elementary steps. Notably, a 2004 experimental study^24^ corroborates the initial Knoevenagel condensation: the coupling of an arylaldehyde, malononitrile, and 4-amino-2,6-dihydroxy pyrimidine afforded the same pyrido­[2,3-*d*]­pyrimidine product obtained from the reaction between 3,4-dichlorophenyl methylidene malononitrile and 4-amino-2,6-dihydroxy pyrimidine.

**Scheme 3:**
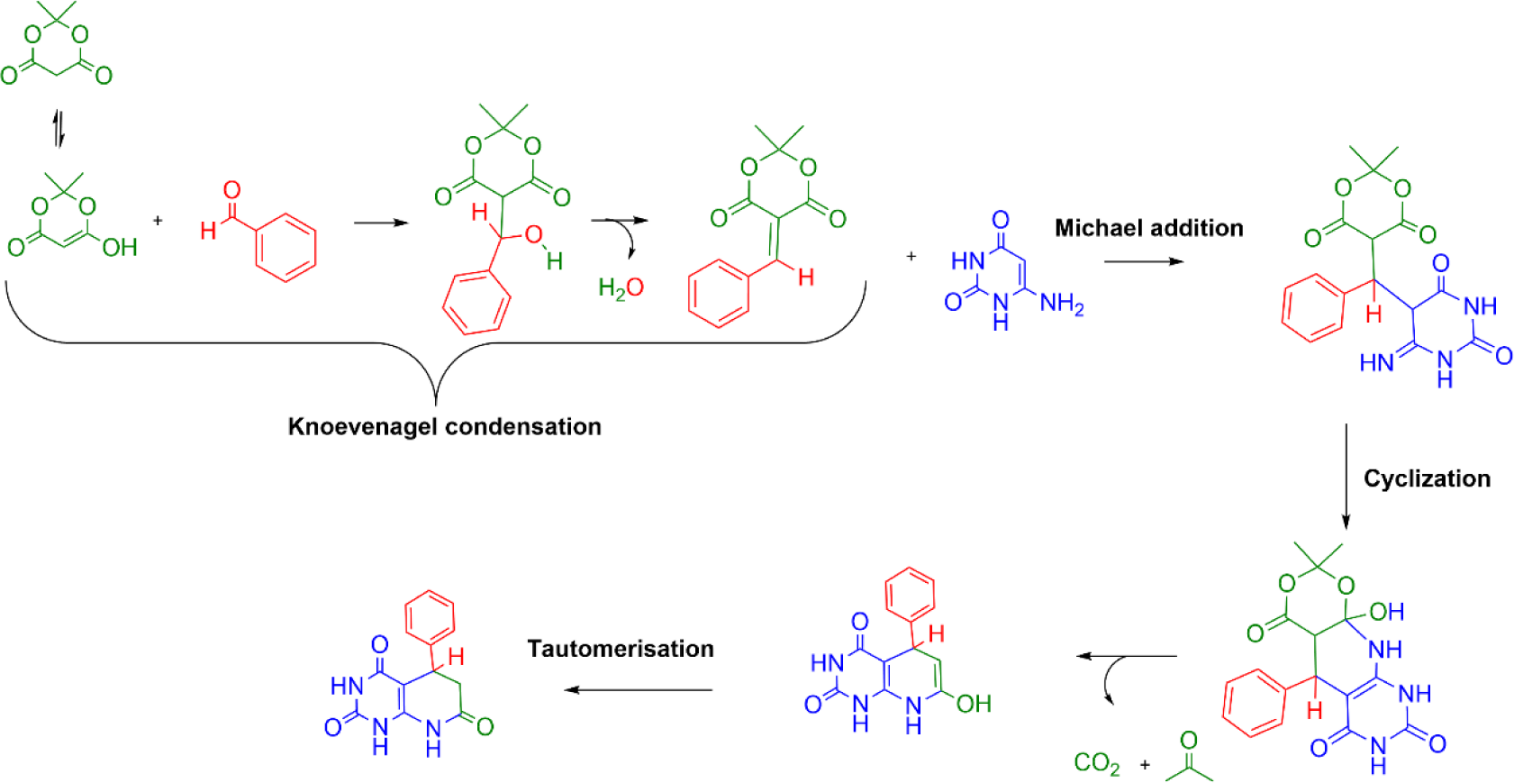
Reaction Mechanism Steps for the Formation of Pyrido­[2,3-*d*]­pyrimidines

To the best of our knowledge, no theoretical investigation has yet addressed the multicomponent mechanisms responsible for the formation of pyrido­[2,3-*d*]­pyrimidines. However, there are indeed computational studies on the classical reactions of Knoevenagel condensation,^25–28^ Michael addition,^29–32^ and cyclization.^33–35^ For the Knoevenagel condensation, Pliego and coworkers^25^ employed the SMD/LPNO-CEPA/1/ma-TZVPP//CPCM/X3LYP/6–31G­(d)­(6-31+G­(d) for O atoms) level of theory and presented two possible mechanisms: a base-catalyzed route and another that begins with keto–enol tautomerism of acetylacetone; the first one exhibited a lower free energy barrier than the second one. Regarding the Michael addition, a study of 2018 ^29^ at the M06–2X/def2-TZVPP (ma-def2-TZVPP for O and N atoms)//SMD/X3LYP/def2-SVP­(ma-def2-SVP for O and N atoms) level of theory indicates that the reaction initiates with an isomerization of the nucleophile, which is followed by the formation of a carbon–carbon bond simultaneously with proton transfer conducted by a bifunctional catalyst, and, by the end, an isomerization of the intermediate leading to the formation of the product. Finally, cyclization has been examined in related multicomponent reactions, such as the Biginelli reaction. In 2015, Morokuma and coworkers^33^ observed that the formation of a bond between nitrogen and the carbonyl carbon occurs simultaneously with the transfer of a proton from the catalyst to the carbonyl oxygen. Such a transition state presented a free energy barrier of 21.5 kcal mol^–1^ at the PCM/M06–2*X*/6–31+G­(d) level of theory.

A complete reaction mechanism elucidation of the formation of pyrido­[2,3-*d*]­pyrimidines is an essential step for comprehending the reaction itself, identifying the rate-determining step, and it is the first step in the search for more effective catalysts for the reaction. The main objective of this work is the theoretical elucidation of the uncatalyzed reaction mechanism among benzaldehyde, Meldrum’s acid, and 6-aminouracil in aqueous solution, while also evaluating the effect of temperature on the reaction.

## Theoretical Methods

### Electronic Structure Calculations

The reaction among benzaldehyde, Meldrum’s acid, and 6-aminouracil in aqueous solution was investigated using theoretical calculations. To obtain accurate free energy values with computational efficiency, we used a sequential method proposed by Simón and Goodman in 2011.^36^ This protocol uses hybrid GGA functionals for geometry optimization and hybrid meta-GGA functionals for single-point energy calculations, the first one allowing an adequate geometry and the second one allowing a more accurate value of electronic energy. The hybrid GGA functional we used was the X3LYP,^37^ which performs better than B3LYP in describing hydrogen bond interactions. The hybrid meta-GGA functional chosen by us for single-point energy calculations was M06–2X,^38^ which has a deviation of 2.6 kcal mol^–1^ for barrier heights.^39^ Since the reaction was conducted in an aqueous medium, the effect of the water solvent was considered using the SMD^40^ solvation model.

In this sense, the geometry optimization and harmonic frequency calculations were performed at the SMD^40^/X3LYP^37^/def2-SVP^41^ level of theory. Single-point energy calculations were performed at the M06–2X^38^/def2-TZVPP^41^ level of theory. Some single-point energy calculations were also performed at the X3LYP/def2-SVP level of theory to obtain the solvation free energy, which is the difference from the previous SMD/X3LYP/def2-SVP energy. Finally, the solution-phase free energy can be obtained by applying eq 1:

1 $$G_{\mathrm{sol}}=E_{\mathrm{el}}+G_{n}+{\Delta G}_{\mathrm{solv}}+\mathrm{SSC}\;\left({\mathrm{kcal mol}}^{-1}\right)$$

where *G*_sol_ is the solution-phase free energy. *E*_el_ refers to the gas-phase electronic energy obtained at the M06–2X/def2-TZVPP level of theory. *G*_n_ refers to thermal corrections to the free energy obtained at the SMD/X3LYP/def2-SVP level of theory. Δ*G*_solv_ is the solvation free energy obtained as the difference between the SMD/X3LYP/def2-SVP and X3LYP/def2-SVP levels of theory. SSC, from 1 atm to 1 mol L^–1^, depends on the temperature and can be calculated by eq 2:

2 $$\mu^{*}=\mu^{{}^{\circ}}+RT\ln\left(\frac{RTC^{*}}{p^{{}^{\circ}}}\right)$$

Where μ^*^ is the chemical potential in the standard state of 1 mol L^–1^, μ^°^ is the chemical potential in the standard state of 1 atm, *R* is the gas constant, *T* is the temperature (between 298 and 398 K), *C* ^*^ is the standard concentration of 1 mol L^–1^, and *p*° is the standard pressure of 1 atm. The SSC value for each temperature analyzed in this work is presented in Table S1. All the calculations were done using the Orca 5.0 program.^42–44^

### Calculation of p*K*_a_

The first mechanistic step of the multicomponent reaction is a Knoevenagel condensation, with the possibility of occurring via a base mechanism.^25^ Considering this possibility, one of the reactants, 6-aminouracil, or the solvent, water, could act as a base, deprotonating Meldrum’s acid. In this context, we have used a proton exchange scheme with reference species for p*K*_a_ calculation in water.^45^ For generation of an anionic species from Meldrum’s acid, we have used PhOH (p*K*_a_ = 9.99)^46^ as a reference (eqs 3 and 4). For generation of cationic species, specifically for the hydronium cation, we used protonated methylamine (p*K*_a_ = 10.64),^46^ and for protonated 6-aminouracil, we used protonated aniline (p*K*_a_ = 4.6)^46^ as a reference species (eqs 5 and 6).

3 $$HA+\mathrm{Ph}O^{-}⇌A^{-}+PhOH$$

4 $$pK_{a}\left(HA\right)=\frac{\Delta G_{sol}}{RT\ln\left(10\right)}+pK_{a}\left(PhOH\right)$$

5 $$BH^{+}+MeNH_{2}\left(or\;PhNH_{2}\right)⇌B+MeNH_{3}^{+}\left(or\;PhNH_{3}^{+}\right)$$

6 $$pK_{a}\left(BH^{+}\right)=\frac{\Delta G_{sol}}{RT\ln\left(10\right)}+pK_{a}\left(MeNH_{3}^{+}\;or\;PhNH_{3}^{+}\right)$$

### Kinetic Analysis and Microkinetic Modeling

We performed a kinetic analysis for the formation of 5-phenyl-5,6-dihydropyrido­[2,3-*d*]­pyrimidine-2,4,7­(1*H,*3*H,*8*H*)-triones in aqueous solution using the rate-determining step for determination of the rate law. We employed the conventional transition state theory for the calculation of kinetic constants:

7 $$k\left(T\right)=\frac{k_{b}T}{h}e^{-\Delta G_{\mathrm{sol}}^{‡}\left(T\right)/RT}$$

where *K*(*T*) is the kinetic constant, *K*_b_ is the Boltzmann constant, *h* is the Planck constant, *R* is the gas constant, *T* is the temperature, and $\Delta G_{sol}^{‡}$ is the activation Gibbs free energy. As can be observed in eq 7, there is a dependence of $\Delta G_{sol}^{‡}$ and the kinetic constant (*K*) on temperature, allowing us to also determine the impact of temperature on the rate-determining step.

Equation 7 was also used to determine the kinetic constants for each step of the reaction mechanism involved in the formation of 5-phenyl-5,6-dihydropyrido­[2,3-*d*]­pyrimidine-2,4,7­(1*H*,3*H*,8*H*)-triones in aqueous solution. For steps where tautomerizations, complexations, decomplexations, and proton exchange processes occur, we used the equilibrium constants for determining the forward and backward kinetic constants:

8 $$K_{eq}=\frac{k_{+}}{k_{-}}$$

Finally, we combine reagents concentrations data from the experimental study with the kinetic constants calculated by us to obtain a more detailed description of the behavior of the reaction over time and with temperature variation, through microkinetic modeling.^47^ All the microkinetic modeling calculations were conducted with the Kintecus program.^48^

## Results and Discussion

### Reaction Mechanisms

In the literature,^22^ it has been suggested that the reaction between benzaldehyde, Meldrum’s acid, and 6-aminouracil occurs through four mechanistic steps: (i) Knoevenagel condensation, (ii) Michael addition, (iii) cyclization, and (iv) propanone and CO_2_ release. Each mechanistic step was studied separately in this work and is presented below.

### Knoevenagel Condensation

The Knoevenagel condensation can occur by two mechanisms: via base^25^ or via tautomerization of Meldrum’s acid (Figure 1). In the first case, a molecule of water or a molecule of 6-aminouracil can act as a base, deprotonating the Meldrum’s acid. We calculated the p*K*_a_ value for each species involved (Meldrum’s acid, 6-aminouracil, H_2_O) utilizing a proton exchange scheme presented in the methodology section. The results of these calculations are presented in Table 1. As can be seen, the lowest p*K*_a_ for a base species is for 6-aminouracil, 8.8, while it is 11.2 for water, which indicates that 6-aminouracil deprotonates Meldrum’s acid more easily than water.^49^ In this context, the base mechanism initiates with a deprotonation of Meldrum’s acid by 6-aminouracil, leading to the formation of Meldrum’s acid anion and protonated 6-aminouracil with a free energy in solution of 12.6 kcal mol^–1^. In the next step, there is the formation of a carbon–carbon bond from the nucleophilic attack of Meldrum’s acid anion on the carbonyl carbon of benzaldehyde. This transition state, named TS1k, has a free energy barrier of 37.2 kcal mol^–1^ and leads to the formation of an anion, MS5a, with a free energy in solution of 35.6 kcal mol^–1^ relative to the reactants. This species can then isomerize into another anionic form, MS5b, with a proton transfer from carbon atom to oxygen atom and a free energy in solution of 12.6 kcal mol^–1^. In the next step, the protonated 6-aminouracil can donate a proton to MS5b, forming the neutral species MS5c, with a free energy in solution of 6.1 kcal mol^–1^. MS5c can isomerize into an enolic form, MS5d, with a solution free energy of 8.7 kcal mol^–1^. In the final step of this mechanism, there is a detachment of a hydroxide ion with simultaneous deprotonation of the hydroxyl group, forming a molecule of water and arylidene (MS4). The transition state for this step, TS2k, has a free energy barrier of 25.9 kcal mol^–1^, and the products, MS4 and water, have a free energy in solution of 0.1 kcal mol^–1^.

**Table 1:** Reaction Thermodynamic Properties for the Knoevenagel Condensation Mechanism^a^

Entry	Process	*W* ^b^	Δ*E*_ele_ ^c^	Δ*G*_n_ ^d^	ΔΔ*G*_solv_ ^e^	Δ*G*_sol_ ^f^	p*K*_a_ ^g^	Δ*G*_desp_ ^h^
1	6-aminouracilH^+^ + PhNH_2_ → 6-aminouracil + PhNH_3_^+^	–16.74	–29.09	–0.06	10.90	–18.25	–8.78	–11.99
2	H_3_O^+^ + CH_3_NH_2_ → H_2_O + CH_3_NH_3_^+^	–29.01	–50.17	1.41	18.99	–29.77	–11.19	–15.27
3	Meldrum’s acid + PhO^–^ → Meldrum’s acid anion + PhOH	–11.48	–20.87	0.55	7.33	–12.99	0.46	0.63
4	Meldrum’s acid + H_2_O → H_3_O^+^ + Meldrum’s acid anion	-	-	-	-	-	´-	15.91
5	Meldrum’s acid + 6-aminouracil → 6-aminouracilH^+^ + Meldrum’s acid anion	-	-	-	-	-	-	12.62

a Units are in kcal mol^–1^. Standard state of 1 mol L^–1^ for all the species.

b Potential of mean force obtained at the SMD/X3LYP/def2-SVP level.

c Electronic energy using the M06–2X/def2-TZVPP basis set.

d Translational, rotational, and vibrational contributions to the free energy.

e Solvent effect (water) obtained at the SMD/X3LYP/def2-SVP level of theory.

f Solution phase free energy.

g p*K*_a_ values.

h Δ*G*_desp_ = 1.364 p*K*_a_.

**Figure 1:**
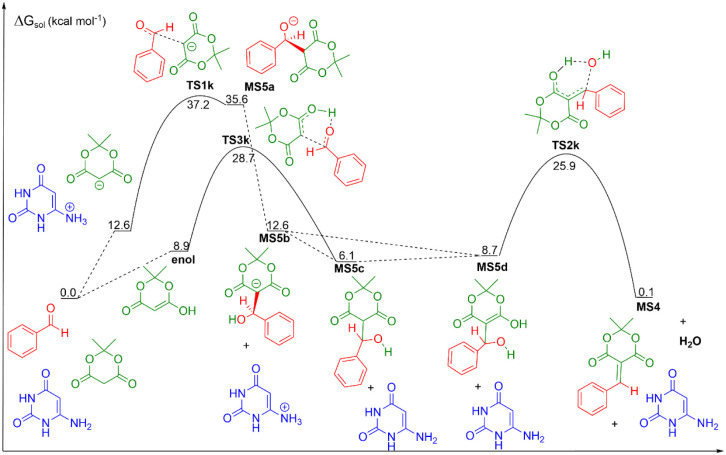
Knoevenagel condensation reaction between benzaldehyde and Meldrum‘s acid, the first mechanistic step in the formation of 5-phenyl-5,6-dihydropyrido­[2,3-*d*]­pyrimidine-2,4,7­(1*H*,3*H*,8*H*)-triones in aqueous solution. Units are in kcal mol^–1^, the standard state of 1 mol L^–1^ for all species at 298 K.

As can be observed, a base mechanism has the first step kinetically infeasible due to the very high barrier (37.2 kcal mol^–1^). Therefore, we have investigated a second mechanistic possibility. In this second mechanism, a keto–enol tautomerism would initially occur, forming the enol form of Meldrum’s acid. The formation of the enol has a free energy in solution of 8.9 kcal mol^–1^ relative to the reagents. The next step would be the nucleophilic attack of the enol on the carbonyl carbon of benzaldehyde, with a simultaneous proton transfer from the hydroxyl group of the enol to the carbonyl oxygen of benzaldehyde. The transition state for this step, TS3k, has a free energy barrier of 28.7 kcal mol^–1^, which can be kinetically feasible if subjected to an increase in temperature, as the reaction is experimentally conducted. The product of this step, MS5c, has a free energy in solution of 6.1 kcal mol^–1^, and it can tautomerize to the enol form, MS5d, with a free energy in solution of 8.7 kcal mol^–1^. As in the previous case, this species goes through a transition state with the release of a water molecule, TS2k, and forms the product MS4. Therefore, our results suggest that the mechanism initiates with a keto–enol tautomerism, which is more feasible than a base mechanism.

### Michael Addition

The reaction follows the next mechanism, a Michael addition (Figure 2). In this case, MS4 has two face attacks, Re and Si. The nucleophilic attack of 6-aminouracil on the Re face of MS4, TS4kA, has a free energy barrier of 19.0 kcal mol^–1^, leading to the formation of an R-intermediate, MS6A, with a solution free energy of 13.4 kcal mol^–1^. On the other hand, the nucleophilic attack of 6-aminouracil on the Si face of MS4, TS4kB, has a free energy barrier of 17.3 kcal mol^–1^ and its S-intermediate, MS6B, has a solution free energy of 10.0 kcal mol^–1^. In sequence, there is a proton transfer from H_2_N– group to the negatively charged oxygen, catalyzed by a molecule of water. For the R-isomer, TS5kA-H_2_O has a free energy in solution of 17.5 kcal mol^–1^, and its respective product, a complex with water, MS7A-H_2_O, has a free energy in solution of 17.1 kcal mol^–1^. The decomplexation of the water molecule is slightly unfavorable with MS7A and water having a solution free energy of 19.9 kcal mol^–1^. So, despite the figure, the transition state (TS5kA-H_2_O) has a free energy lower than MS7A; however , its high barrier is above the energy of its respective product, MS7A-H_2_O. For the S-isomer, TS5kB-H_2_O has a free energy barrier of 16.1 kcal mol^–1^ and its complex product, MS7B-H_2_O, has a solution free energy of 17.7 kcal mol^–1^. The decomplexation of MS7B-H_2_O, to MS7B and water has a solution free energy of 15.9 kcal mol^–1^, which indicates that its decomplexed form is more stable than the complexed form. Finally, MS7A and MS7B, both in enol form, tautomerize to the keto form, MS8A and MS8B, with solution free energies of 10.5 and 10.6 kcal mol^–1^, respectively. It is important to note that, when the tautomerization occurs, there is a change in the priority groups, implying an inversion of the enantiomers: MS8A is now an (*S*)-isomer and MS8B is an (*R*)-isomer. The second point to clarify is that, in the absence of a chiral catalyst to promote asymmetric induction, the reaction would not proceed enantioselectively. We merely point out that, if such a catalyst were employed, the reaction could potentially proceed with enantioselectivity.

**Figure 2:**
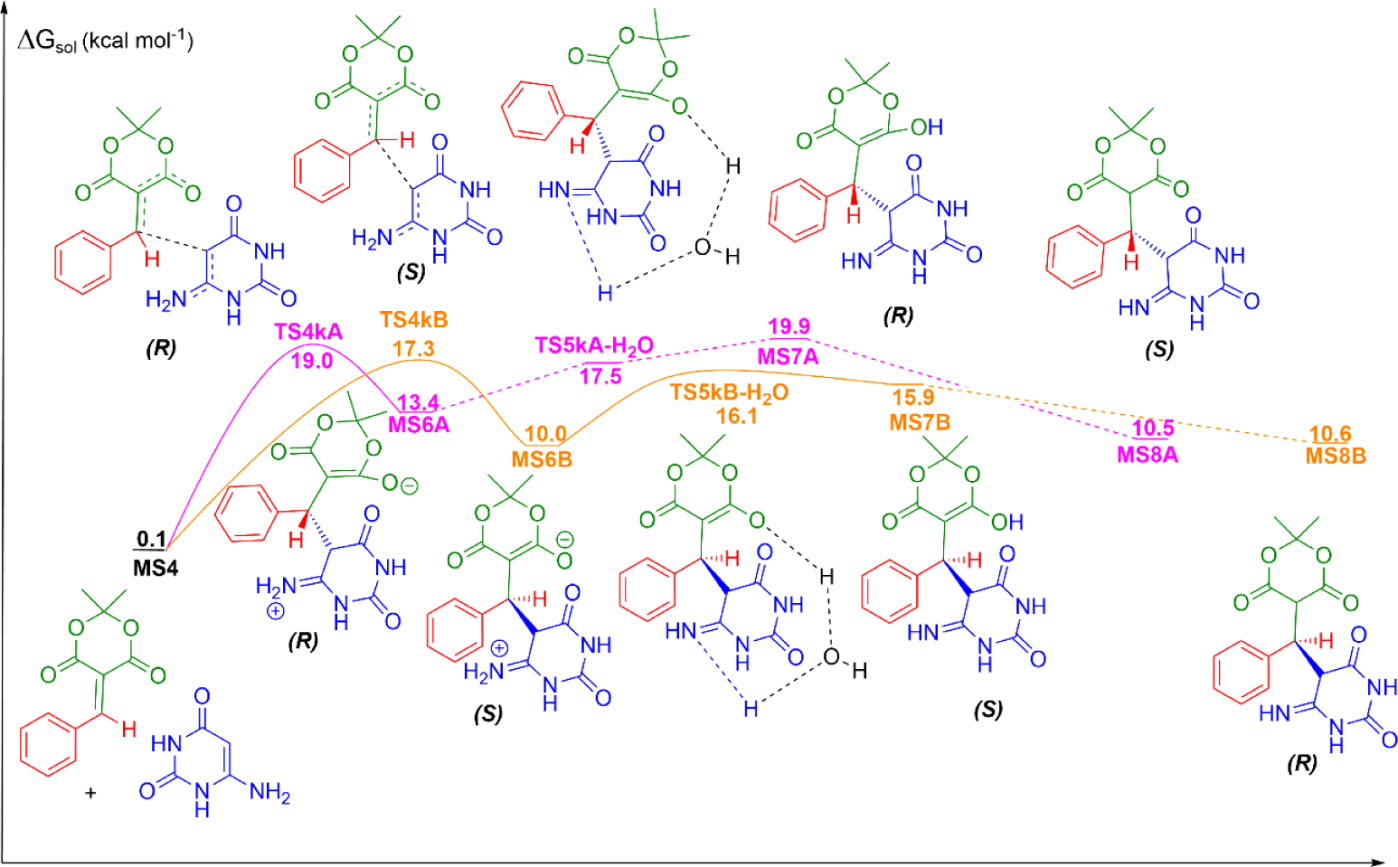
Michael addition reaction among benzaldehyde, Meldrum’s acid, and 6-aminouracil, the second mechanistic step in the formation of 5-phenyl-5,6-dihydropyrido­[2,3-*d*]­pyrimidine-2,4,7­(1*H*,3*H*,8*H*)-triones in aqueous solution. Units are in kcal mol^–1^, with the standard state of 1 mol L^–1^ for all species at 298 K.

### Cyclization

The next reaction mechanism is a cyclization, through the nucleophilic attack of a nitrogen atom from the imine group on one of the carbonyl carbons, generating two possibilities from each species, MS8A and MS8B (Figure 3). Considering MS8A initially, one of the nucleophilic attacks generates the diastereomer (*S,S*), named MS10A1, with a solution free energy of 22.8 kcal mol^–1^. The corresponding transition state, TS8A1, has a free energy barrier of 23.5 kcal mol^–1^. On the other hand, the second possibility of nucleophilic attack, with MS8A as the initial structure, generates the diastereomer (*S,R*), named MS10A2, with a solution free energy of 21.6 kcal mol^–1^, and its transition state, TS8A2, has a free energy barrier of 24.1 kcal mol^–1^. For MS8B, there should also be two possibilities of nucleophilic attack: one generating the diastereomer (*R,S*) and another (*R,R*). In the first case, we located a transition state, TS8B1, with a free energy barrier of 22.1 kcal mol^–1^, which connects to a stable structure, MS10B1, with a solution free energy of 20.6 kcal mol^–1^. In the second case, any attempt to optimize the geometry of MS10B2 dissociated the bond between the carbon and nitrogen atoms, yielding MS8B, which indicates that structure MS10B2 is not a minimum point on the potential energy surface. Therefore, there is the formation of three diastereomers: (*S,S*) (MS10A1), (*S,R*) (MS10A2), and (*R,S*) (MS10B1). In the next step, a proton transfer occurs from the tertiary carbon of the uracil group to the negatively charged oxygen atom, which generates the diastereomers MS11A1 (*S,S*), MS11A2 (*S,R*), and MS11B1 (*R,S*), with solution free energies of 4.6, −2.2, and −1.5 kcal mol^–1^, respectively.

**Figure 3:**
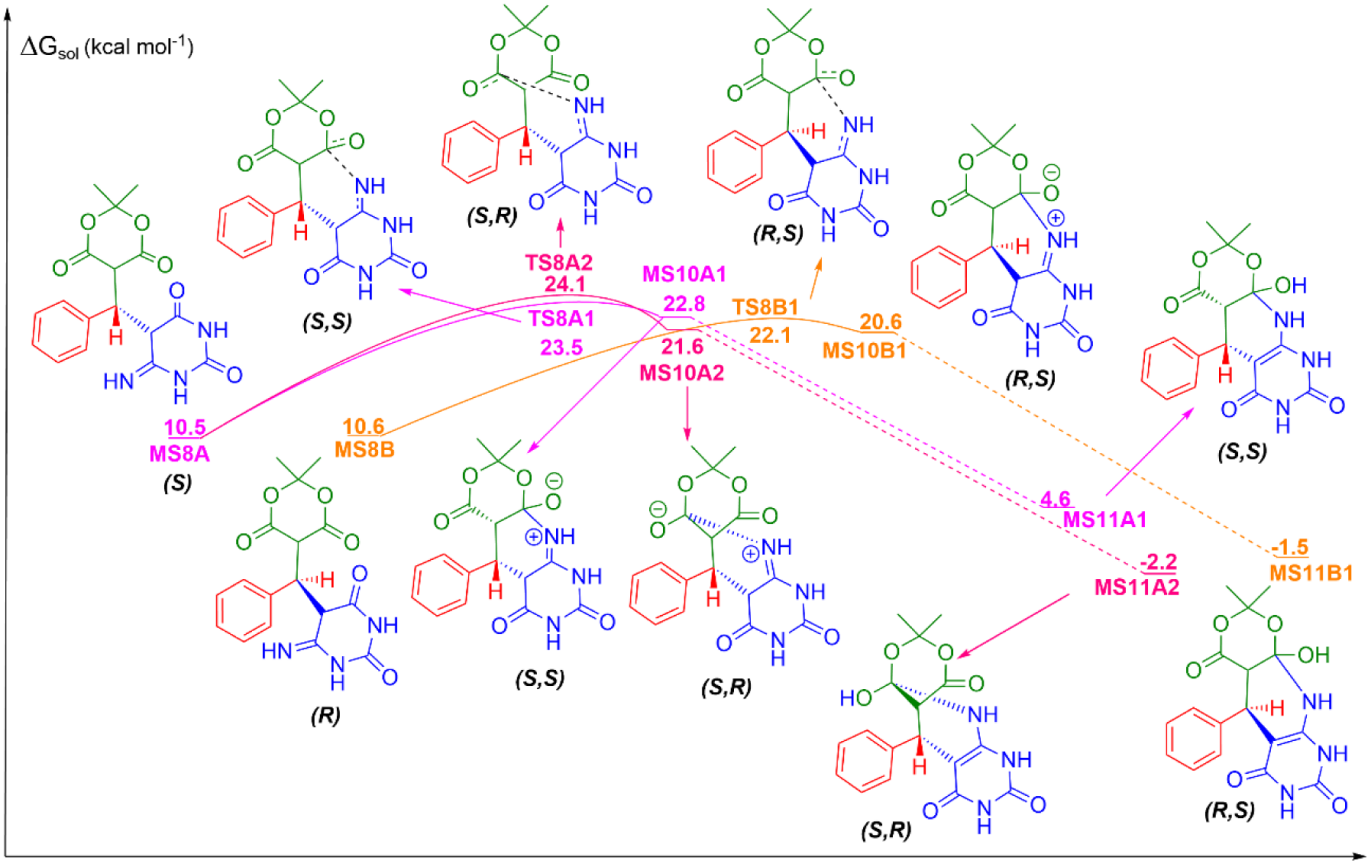
Cyclization reaction among benzaldehyde, Meldrum’s acid, and 6-aminouracil, the third mechanistic step in the formation of 5-phenyl-5,6-dihydropyrido­[2,3-*d*]­pyrimidine-2,4,7­(1*H*,3*H*,8*H*)-triones in aqueous solution. Units are in kcal mol^–1^, with the standard state of 1 mol L^–1^ for all species at 298 K.

### Propanone Release

The next step of the mechanism is the release of a propanone molecule from each diastereomer MS11 (A1, A2, and B1) (Figure 4). In the transition state structure, we observe the cleavage of two bonds: one between a primary carbon and an oxygen atom, and the second between a secondary carbon and an oxygen atom, generating a molecule of propanone and a zwitterion named MS13A1, MS13A2, or MS13B1. The transition state structures for the formation of this species, TS10A1, TS10A2, and TS10B1, have a free energy barrier of 27.7, 25.3, and 25.5 kcal mol^–1^, respectively, and the minimal structures, MS13A1, MS13A2, and MS13B1, have solution free energies of 7.7, 2.8, and 3.3 kcal mol^–1^, respectively.

**Figure 4:**
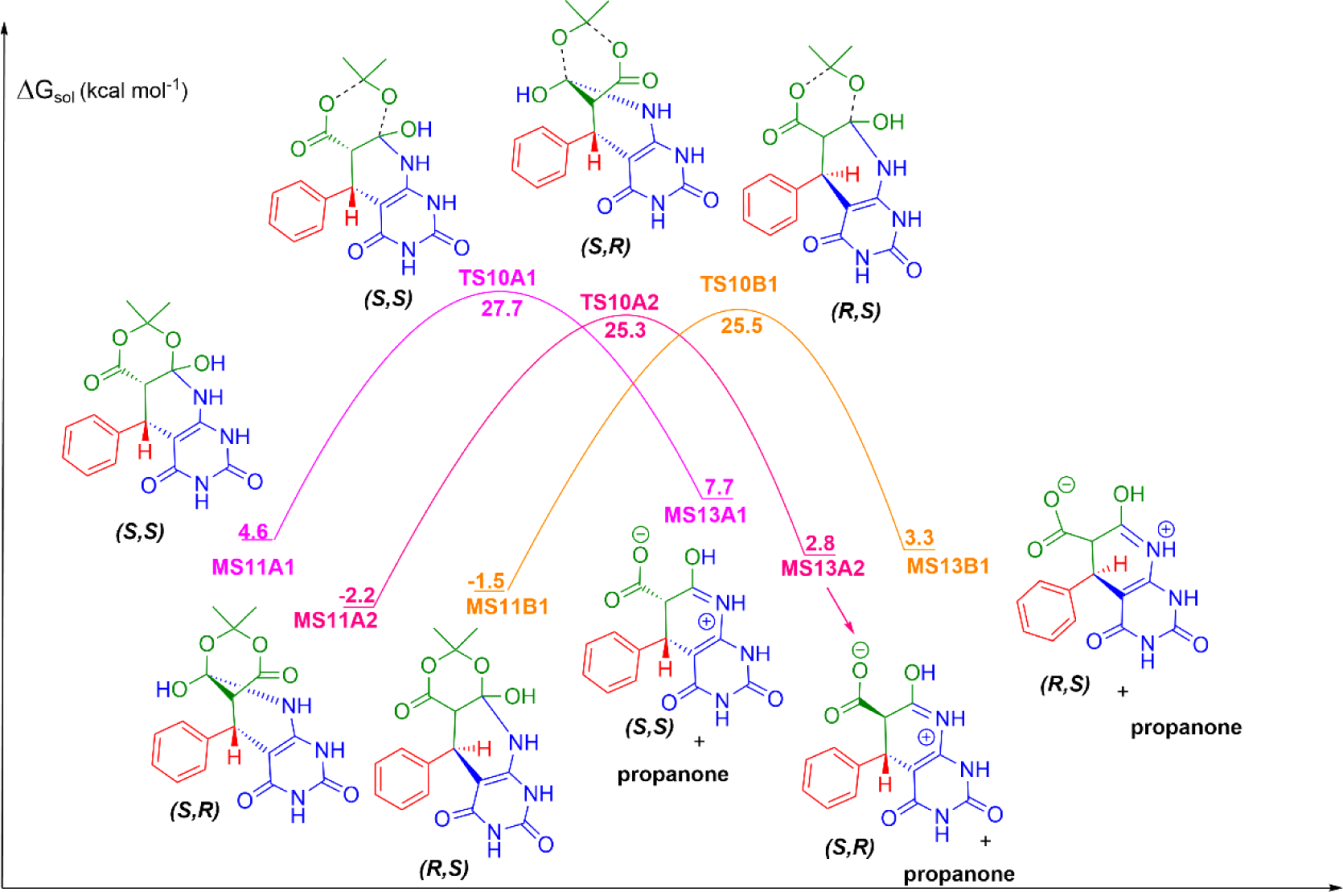
Propanone release from the reaction among benzaldehyde, Meldrum’s acid, and 6-aminouracil, the fourth mechanistic step in the formation of 5-phenyl-5,6-dihydropyrido­[2,3-*d*]­pyrimidine-2,4,7­(1*H*,3*H*,8*H*)-triones in aqueous solution. Units are in kcal mol^–1^, with the standard state of 1 mol L^–1^ for all species at 298 K.

### CO_2_ Release

In the last step of the reaction mechanism, we observe the release of a CO_2_ molecule and the formation of an enol tautomer of the final product, from the cleavage of the bond between two carbon atoms of the zwitterion intermediate (Figure 5). The respective transition states are TS11A1, TS11A2, and TS11B1, with free energy barriers of 10.9, 8.4, and 7.4 kcal mol^–1^, respectively. Through these transition states, there is the formation of the enol tautomers of the final product, MS15A1, MS15A2, and MS15B1, with solution free energies of −11.5, −11.5, and −11.8 kcal mol^–1^, respectively. In the final step of this reaction, there is a keto–enol tautomerism, generating the final product in its keto form, MS16A1, MS16A2, and MS16B1, with solution free energies of −27.9, −29.5, and −29.9 kcal mol^–1^.

**Figure 5:**
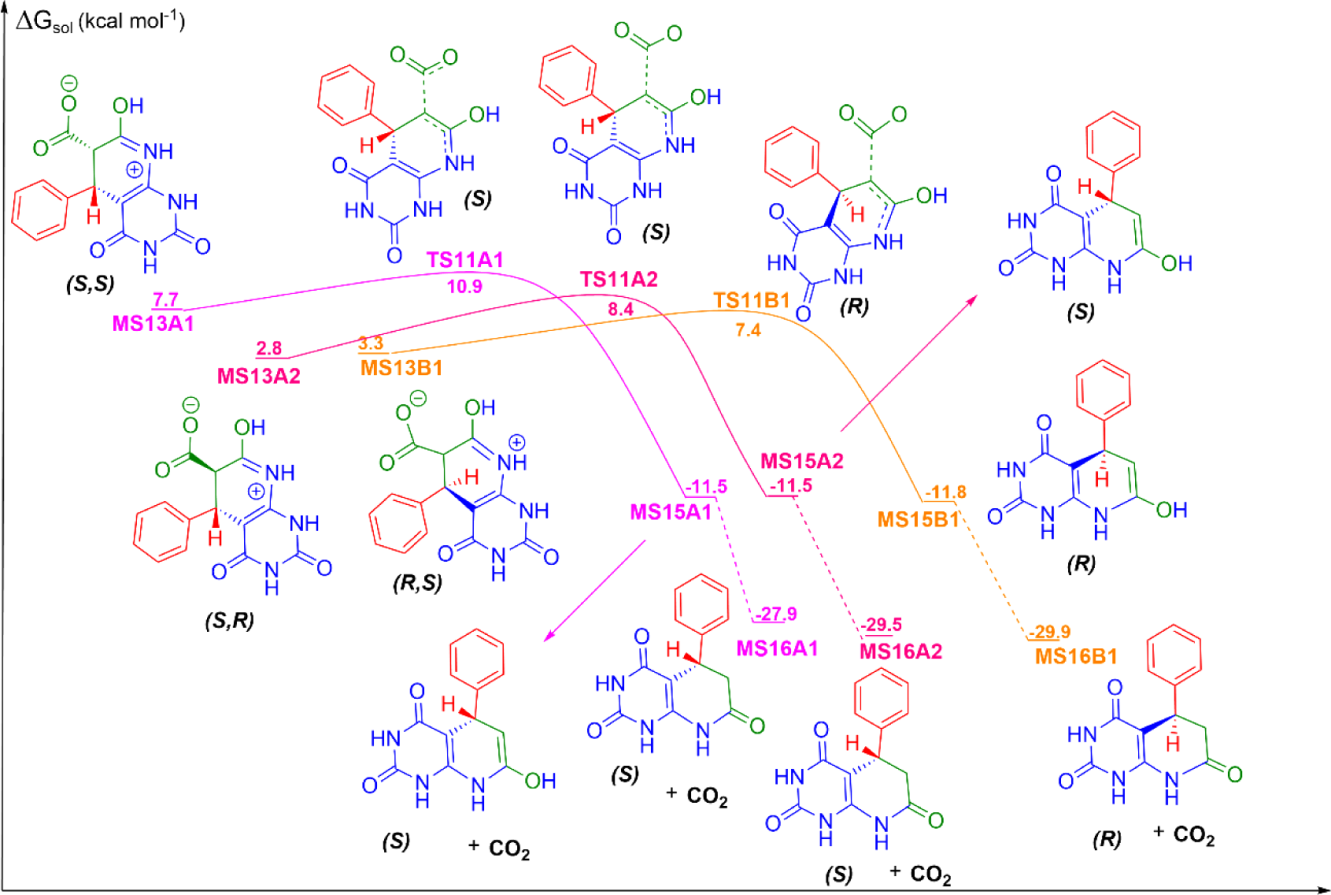
CO_2_ release from the reaction among benzaldehyde, Meldrum’s acid, and 6-aminouracil, the fifth mechanistic step in the formation of 5-phenyl-5,6-dihydropyrido­[2,3-*d*]­pyrimidine-2,4,7­(1*H*,3*H*,8*H*)-triones in aqueous solution. Units are in kcal mol^–1^, with the standard state of 1 mol L^–1^ for all species at 298 K.

### Kinetic Analysis

Because the reaction was experimentally conducted under reflux conditions, we evaluated the impact of a gradual temperature increase in 10 K intervals, starting from 298 K. In Table S3, we present the solution-phase free energy as a function of temperature for every species studied here, and in Table 2, the activation free energy and kinetic constants for the rate-determining step. In both tables, we observe that there is a gradual increase in the value of solution-phase free energy with the increase of temperature. From a kinetic viewpoint, the increase of temperature has also resulted in an increase in the rate constant and will be explored in sequence.

**Table 2:** Activation Free Energy and Rate Constants for the Reaction between Benzaldehyde, Meldrum’s Acid, and 6-Aminouracil in Aqueous Solution, Considering the Rate-Determining Step (TS3k)

Temperature (K)	Δ*G* ^‡^ (kcal mol^–1^)	*k* (L^2^ mol^–2^ s^–1^)
**298**	28.75	5.08 × 10^–9^
**308**	29.18	1.26 × 10^–8^
**318**	29.62	2.90 × 10^–8^
**328**	30.04	6.56 × 10^–8^
**338**	30.48	1.37 × 10^–7^
**348**	30.92	2.76 × 10^–7^
**358**	31.36	5.33 × 10^–7^
**368**	31.79	1.01 × 10^–6^
**378**	32.23	1.82 × 10^–6^
**388**	32.65	3.28 × 10^–6^
**398**	33.10	5.51 × 10^–6^

As can be observed in Figure 1, the first step, with the formation of a carbon–carbon bond between benzaldehyde and the tautomeric enol of Meldrum’s acid in TS3k, is the rate-determining step. From the species involved in this step, we can define the following rate law:

$$\frac{d\left[MS5c\right]}{dt}=k\left[Meldrum^{\prime }s\;acid\right]\left[benzaldehyde\right]$$

The transition state TS3k leads to a free energy barrier of 28.7 kcal mol^–1^ at 298 K, which corresponds to a rate constant of 5.08 × 10^–9^ L^2^ mol^–2^ s^–1^. The kinetic constants for the additional temperatures are presented in Table 2, and as can be seen, from 298 to 398 K, the rate constant has increased by about a thousand (10^3^) times. Consequently, although the increase in temperature thermodynamically has unfavored the reaction with the higher barriers, from a kinetic point of view, there was a significant increase in the reaction rates.

### Microkinetic Analysis

We carried out a microkinetic analysis of the reaction between benzaldehyde, Meldrum’s acid, and 6-aminouracil in aqueous solution, which was carried out experimentally at reflux conditions (373 K), with a concentration of 1 mol L^–1^ for all the reagents and a reaction time of 10 h.^22^ As mentioned earlier, we evaluated the effect of temperature on the reaction kinetics. In the present analysis, we considered two temperatures: 298 and 368 K, the temperature close to the boiling point of water. The microkinetic model used in this study is presented in the Supporting Information, whose kinetic constants (Table S4) were calculated using transition state theory and the kinetic equation integrated with the Kintecus program.

The results of microkinetic modeling are presented in Figure 6a,b. In both analyses, we have considered two axes for the concentration species: the primary axis for Meldrum’s acid and the secondary axis for MS16B1, one of the stereoisomer intermediates formed. These graphics are presented in this way because the product is formed in only trace quantities, which is difficult to visualize. In both Figure 6a,b, we can observe the decline of Meldrum’s acid concentration, one of the reagents, and an increase in the concentration of MS16B1. The main difference between these two figures is MS16B1 concentration, which, over the same reaction time of 10 h, increases from 4.52 × 10^–9^ to 3.99 × 10^–5^ mol L^–1^. These theoretical results are in good agreement with the experimental results, where under reflux conditions, only traces of the intermediate are observed.^22^

**Figure 6:**
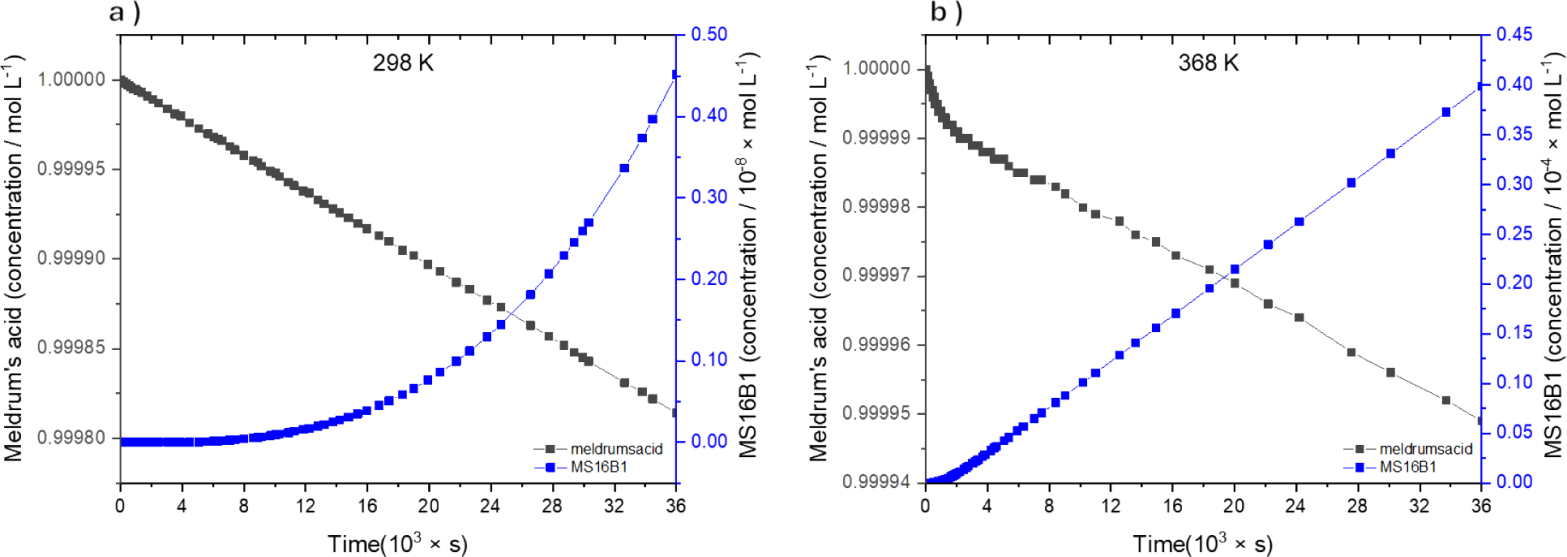
Microkinetic modeling of the reaction among benzaldehyde (1 mol L^–1^), Meldrum’s acid (1 mol L^–1^), and 6-aminouracil (1 mol L^–1^) in aqueous solution, based on theoretical rate constants and 10 h of reaction time at a) 298 K and b) 368 K.

## Conclusions

In this work, we have conducted a theoretical investigation of the reaction mechanism for the formation of pyrido­[2,3-*d*]­pyrimidines in aqueous solution. From our results, the mechanism occurs through keto/enol tautomerism, the Knoevenagel condensation, Michael addition, cyclization, propanone release, CO_2_ release, and steps with tautomerization and/or proton transfer processes. We also considered a base mechanism for the Knoevenagel condensation, but this was kinetically unfeasible. The rate-determining step is the nucleophilic attack from the enol tautomer of Meldrum’s acid to the carbonylic carbon of benzaldehyde, whose very high energy barrier at room temperature justifies the kinetic unfeasibility in the absence of a catalyst. The increase in temperature has a significant impact on the reaction kinetics, which could be observed in the microkinetic modeling, even so, allowing only the formation of traces of the product, in good agreement with the experimental results. To the best of our knowledge, this consists of the first complete theoretical elucidation of a reaction mechanism for the formation of pyrido­[2,3-*d*]­pyrimidines in aqueous solutions. The rate-determining step identified in this study can steer researchers toward designing more efficient catalysts, especially those that lower the Gibbs free energy barrier of the Knoevenagel condensation. In addition, our results indicated that the reaction can be conducted in an enantioselective manner; however, this requires the presence of a chiral catalyst in the reaction medium.

## Supplementary Material



## Notes

Standard state correction (Table S1); reaction and activation thermodynamic properties (Table S2); reaction and activation solution-phase free energy at different temperatures (Table S3); activation thermodynamic properties and rate constants; microkinetic model equations (Table S4); and Cartesian coordinates for all stationary points on the potential energy surface (PDF)
