# Supplementary material for: Aqueous-Phase Multicomponent Reaction Mechanism for the Synthesis of Pyrido[2,3‑d]pyrimidines: A Theoretical Perspective
Source: ACS Omega. 2025 Jun 19;10(25):27393–403. doi: 10.1021/acsomega.5c03056 (PMC12223879; doi:10.1021/acsomega.5c03056)
Supplement: Supplementary file 1 [file ao5c03056_si_001.pdf]

Aqueous-Phase Multicomponent Reaction Mechanism for the Synthesis of Pyrido[2,3-d]pyrimidines: A Theoretical Perspective

Virginia C. Rufino, Giovanni W. Amarante and Hélio F. Dos Santos

Department of Chemistry, Federal University of Juiz de Fora, Rua José Lourenço Kelmer, Campus Universitário São Pedro, Juiz de Fora, Minas Gerais 36036-900, Brazil.

Table S1. Standard state correction (SSC) values as a function of temperature.

Temperature (K)	SSC (kcal mol^{-1})
298	1.89
308	1.97
318	2.06
328	2.15
338	2.23
348	2.32
358	2.40
368	2.49
378	2.58
388	2.67
398	2.75

Table S2: Reaction and activation thermodynamic properties for the reaction between benzaldehyde, Meldrum's acid and 6-aminouracil in aqueous solution.[a]

	**Knoevenagel condensation**					
	**Base mechanism**					
**Entry**	**process**	**W[b]**	**M06-2X[c]**	**ΔGn[d]**	**$\Delta\Delta G_{solv}$[e]**	**ΔG_{sol}[f]**
1	Meldrum's acid + H_2O → H_3O^+ + Meldrum's acid anion	-	-	-	-	15.91
2	Meldrum's acid + 6-aminouracil → 6-aminouracilH$^+$ + Meldrum's acid anion	-	-	-	-	12.62
3	Meldrum's acid anion + benzaldehyde → TS1k	12.62	5.82	13.03	5.69	24.54
4	Meldrum's acid anion + benzaldehyde → MS5a	12.48	7.10	13.27	2.57	22.94
5	Meldrum's acid anion + benzaldehyde → MS5b	-9.99	-23.45	14.94	8.46	-0,05
6	Meldrum's acid + benzaldehyde → MS5d	0.06	-8.41	15.12	2.02	8.73
7	Meldrum's acid + benzaldehyde → TS2k	17.16	18.28	13.20	-5.57	25.92
8	Meldrum's acid + benzaldehyde → MS4 + H_2O	4.32	-0.42	2.76	-2.23	0.11
	**Knoevenagel condensation**					
	**Mechanism via enol**					
**Entry**	**process**	**W[b]**	**M06-2X[c]**	**ΔGn[d]**	**$\Delta\Delta G_{solv}$[e]**	**ΔG_{sol}[f]**
9	Meldrum's acid → enol	10.35	9.20	0.07	-0.41	8.87
10	Meldrum's acid + benzaldehyde → TS3k	16.22	15.36	13.07	0.32	28.75
11	Meldrum's acid + benzaldehyde → MS5c	-3.45	-8.16	15.71	-1.43	6.11

12	Meldrum's acid + benzaldehyde → MS5d	0.06	-8.41	15.12	2.02	8.73
13	Meldrum's acid + benzaldehyde → TS2k	17.16	18.28	13.20	-5.57	25.92
14	Meldrum's acid + benzaldehyde → MS4 + H_2O	4.32	-0.42	2.76	-2.23	0.11
	**Michael addition**					
**Entry**	**process**	**W**[b]	**M06-2X**[c]	**ΔGn**[d]	**$\Delta\Delta G_{solv}$**[e]	**ΔG_{sol}**[f]
15	MS4 + 6-aminouracil→ TS4kA	8.25	10.45	14.83	-6.24	19.03
16	MS4 + 6-aminouracil→ MS6A	2.93	20.04	16.10	-22.72	13.42
17	MS4 + 6-aminouracil→ TS4kB	6.69	8.83	14.41	-5.95	17.29
18	MS4 + 6-aminouracil→ MS6B	-0.07	12.29	16.08	-18.32	10.05
19	MS4 + 6-aminouracil + H2O → MS6A-H2O	-13.38	-5.84	26.41	-6.89	13.68
20	MS4 + 6-aminouracil + H2O → MS6B-H2O	-14.16	-11.48	26.22	-4.49	10.25
21	MS4 + 6-aminouracil + H2O → TS5kA-H2O	-5.58	-9.45	24.31	2.65	17.51
22	MS4 + 6-aminouracil + H2O → MS7A-H2O	-5.99	-17.46	26.23	8.35	17.11
23	MS4 + 6-aminouracil + H2O → TS5kB-H2O	-4.54	-14.29	24.14	6.24	16.10
24	MS4 + 6-aminouracil + H2O → MS7B-H2O	-5.17	-18.17	26.49	9.42	17.74
25	MS4 + 6-aminouracil→ MS7A	12.41	-1.06	17.35	3.60	19.90
26	MS4 + 6-aminouracil→ MS7B	9.04	-8.20	16.50	7.61	15.92
27	MS4 + 6-aminouracil→ MS8A	3.05	-11.52	16.89	5.17	10.53
28	MS4 + 6-aminouracil→ MS8B	4.46	-12.02	16.49	6.09	10.56
	**Cyclisation**					
**Entry**	**process**	**W**[b]	**M06-2X**[c]	**ΔGn**[d]	**$\Delta\Delta G_{solv}$**[e]	**ΔG_{sol}**[f]
29	MS4 + 6-aminouracil→ TS8A1	15.40	10.70	16.89	-4.09	23.50
30	MS4 + 6-aminouracil→ MS10A1	15.39	11.41	16.32	-4.95	22.79
31	MS4 + 6-aminouracil→ TS8A2	15.03	7.68	16.66	-0.23	24.12
32	MS4 + 6-aminouracil→ MS10A2	13.84	10.99	17.05	-6.47	21.57
33	MS4 + 6-aminouracil→ TS8B1	13.72	7.08	16.93	-1.93	22.09
34	MS4 + 6-aminouracil→ MS10B1	12.85	10.88	17.10	-7.38	20.59
35	MS4 + 6-aminouracil→ MS11A1	-1.55	-12.11	17.47	-0.79	4.57
36	MS4 + 6-aminouracil→ MS11A2	-8.61	-18.46	16.86	-0.57	-2.18
37	MS4 + 6-aminouracil→ MS11B1	-8.62	-18.65	17.59	-0.41	-1.47

Entry	Propanone release<br>process	W[b]	M06-2X[c]	ΔGn[d]	$\Delta\Delta G_{solv}$[e]	ΔG_{sol}[f]
38	MS4 + 6-aminouracil→ TS10A1	23.81	43.31	13.38	-28.99	27.70
39	MS4 + 6-aminouracil→ MS12A1	6.98	35.18	11.60	-31.53	15.26
40	MS4 + 6-aminouracil→ MS13A1 + propanone	11.28	38.87	0.85	-32.00	7.72
41	MS4 + 6-aminouracil→ TS10A2	20.36	37.37	13.49	-25.59	25.27
42	MS4 + 6-aminouracil→ MS12A2	1.27	21.61	11.16	-23.91	8.86
43	MS4 + 6-aminouracil→ MS13A2 + propanone	5.64	31.60	0.18	-28.94	2.85
44	MS4 + 6-aminouracil→ TS10B1	20.42	37.32	13.60	-25.44	25.47
45	MS4 + 6-aminouracil→ MS12B1	-9.61	10.92	11.76	-22.18	0.50
46	MS4 + 6-aminouracil→ MS13B1 + propanone	5.56	31.61	0.55	-28.88	3.28

Entry	CO_2 release<br>Process	W[b]	M06-2X[c]	ΔGn[d]	$\Delta\Delta G_{solv}$[e]	ΔG_{sol}[f]
47	MS13A1 → TS11A1	5.58	-16.86	-2.25	22.32	3.21
48	MS13A1 → MS14A1	-9.69	-45.76	-3.83	34.88	-14.71
49	MS13A1 → MS15A1 + CO_2	-5.54	-40.50	-13.34	34.64	-19.20
50	MS13A1 → MS16A1 + CO_2	-26.88	-61.61	-12.29	38.25	-35.64
51	MS13A2 → TS11A2	6.34	-14.14	-0.82	20.47	5.52
52	MS13A2 → MS14A2	-4.05	-38.49	-3.16	31.82	-9.83
53	MS13A2 → MS15A2 + CO_2	0.10	-33.23	-12.74	31.58	-14.40
54	MS13A2 → MS16A2 + CO_2	-23.23	-56.71	-11.59	35.51	-32.79
55	MS13A1 → TS11B1	6.48	-14.14	-2.28	20.51	4.09
56	MS13A1 → MS14B1	-3.34	-37.71	-4.48	31.63	-10.57
57	MS13A1 → MS15B1 + CO_2	-0.04	-33.73	-13.26	31.89	-15.10
58	MS13A1 → MS16B1 + CO_2	-23.21	-56.71	-11.89	35.44	-33.16

[a] Units in kcal mol^{-1}. Standard state of 1 mol L^{-1} for all the species. [b] Potential of mean force, obtained at SMD/X3LYP/def2-SVP level. [c] Electronic energy, using M06-2X/def2-TZVPP basis set. [d] Translational, rotational and vibrational contributions to the free energy. [e] Solvent effect (water) obtained at SMD/X3LYP/def2-SVP level of theory. [f] Solution phase free energy.

Table S3: Reaction and Activation solution-phase free energy for the reaction between benzaldehyde, meldrum's acid and 6-aminouracil in aqueous solution in different temperatures.

	Temperature (K)										
**Process / ΔG_{sol} (kcal/mol)**	**298**	**308**	**318**	**328**	**338**	**348**	**358**	**368**	**378**	**388**	**398**

Meldrum's acid → enol	8.87	8.87	8.87	8.86	8.86	8.86	8.85	8.85	8.85	8.84	8.84
Meldrum's acid + benzaldehyde → TS3k	28.75	29.18	29.62	30.04	30.48	30.92	31.36	31.79	32.23	32.65	33.10
Meldrum's acid + benzaldehyde → MS5c	6.11	6.54	6.98	7.40	7.84	8.27	8.72	9.14	9.58	10.00	10.45
Meldrum's acid + benzaldehyde → MS5d	8.73	9.16	9.59	10.01	10.44	10.87	11.31	11.73	12.16	12.57	13.01
Meldrum's acid + benzaldehyde → TS2k	25.92	26.33	26.75	27.15	27.57	27.98	28.41	28.81	29.23	29.63	30.05
Meldrum's acid + benzaldehyde → MS4 + H_2O	0.11	0.22	0.34	0.44	0.55	0.66	0.78	0.89	1.00	1.10	1.21
MS4 + 6-aminouracil→ TS4kA	19.03	19.51	19.97	20.45	20.92	21.40	21.88	22.35	22.82	23.30	23.79
MS4 + 6-aminouracil→ MS6A	13.42	13.89	14.33	14.80	15.27	15.73	16.20	16.66	17.12	17.58	18.05
MS4 + 6-aminouracil→ TS4kB	17.29	17.76	18.21	18.68	19.15	19.61	20.08	20.55	21.01	21.48	21.96
MS4 + 6-aminouracil→ MS6B	10.05	10.52	10.98	11.45	11.92	12.39	12.87	13.34	13.81	14.28	14.76
MS4 + 6-aminouracil + H2O → MS6A-H2O	13.68	14.42	15.16	15.92	16.67	17.42	18.18	18.92	19.67	20.42	21.20
MS4 + 6-aminouracil + H2O → MS6B-H2O	10.25	11.00	11.73	12.49	13.24	13.98	14.74	15.48	19.22	16.97	17.74
MS4 + 6-aminouracil + H2O → TS5kA-H2O	17.51	18.29	19.06	19.85	20.64	21.43	22.22	23.00	23.79	24.57	25.39
MS4 + 6-aminouracil + H2O → MS7A-H2O	17.11	17.87	18.61	19.37	20.13	20.89	21.65	22.41	23.16	23.92	24.70
MS4 + 6-aminouracil + H2O → TS5kB-H2O	16.10	16.88	17.65	18.43	19.22	19.99	20.78	21.56	22.34	23.12	23.93
MS4 + 6-aminouracil + H2O → MS7B-H2O	17.74	18.50	19.25	20.01	20.78	21.53	22.30	23.05	23.81	24.57	25.36
MS4 + 6-aminouracil→ MS7A	19.90	20.38	20.85	21.33	21.81	22.29	22.77	23.24	23.72	24.20	24.69
MS4 + 6-aminouracil→ MS7B	15.92	16.41	16.87	17.36	17.84	18.33	18.81	19.29	19.78	20.26	20.76
MS4 + 6-aminouracil→ MS8A	10.53	11.01	11.47	11.95	12.43	12.91	13.39	13.87	14.34	14.83	15.32
MS4 + 6-aminouracil→ MS8B	10.56	11.03	11.49	11.97	12.44	12.92	13.40	13.87	14.34	14.82	15.31
MS4 + 6-aminouracil→ TS8A1	23.50	24.02	24.53	25.06	25.58	26.10	26.63	27.15	27.68	28.21	28.75
MS4 + 6-aminouracil→ MS10A1	22.79	23.28	23.75	24.24	24.73	25.22	25.71	26.20	26.69	27.18	27.68
MS4 + 6-aminouracil→ TS8A2	24.12	24.64	25.15	25.67	26.19	26.72	27.24	27.76	28.29	28.81	29.35

MS4 + 6-aminouracil→ MS10A2	21.57	22.07	22.56	23.07	23.57	24.07	24.58	25.08	25.58	26.09	26.61
MS4 + 6-aminouracil→ TS8B1	22.09	22.61	23.12	23.65	24.17	24.70	25.23	25.75	26.28	26.81	27.35
MS4 + 6-aminouracil→ MS10B1	20.59	21.10	21.59	22.10	22.61	23.12	23.63	24.13	24.64	25.15	25.67
MS4 + 6-aminouracil→ MS11A1	4.57	5.07	5.55	6.05	6.54	7.04	7.53	8.02	8.52	9.01	9.52
MS4 + 6-aminouracil→ MS11A2	-2.18	-1.69	-1.23	-0.74	-0.26	0.22	0.71	1.19	1.67	2.15	2.65
MS4 + 6-aminouracil→ MS11B1	-1.47	-0.97	-0.49	0.02	0.51	1.01	1.51	2.01	2.51	3.01	3.52
MS4 + 6-aminouracil→ TS10A1	27.70	28.16	28.59	29.05	29.50	29.95	30.41	30.86	31.31	31.76	32.23
MS4 + 6-aminouracil→ MS12A1	15.26	15.65	16.02	16.42	16.80	17.19	17.58	17.96	18.35	18.74	19.14
MS4 + 6-aminouracil→ MS13A1 + propanone	7.72	7.76	7.80	7.85	7.89	7.94	7.98	8.02	8.07	8.12	8.17
MS4 + 6-aminouracil→ TS10A2	25.27	25.72	26.16	26.61	27.06	27.51	27.96	28.41	28.86	29.31	29.77
MS4 + 6-aminouracil→ MS12A2	8.86	9.24	9.60	9.99	10.37	10.75	11.13	11.51	11.89	12.27	12.67
MS4 + 6-aminouracil→ MS13A2 + propanone	2.85	2.88	2.91	2.95	2.98	3.02	3.05	3.09	3.13	3.17	3.21
MS4 + 6-aminouracil→ TS10B1	25.47	25.93	26.36	26.82	27.27	27.73	28.18	28.63	29.09	29.54	30.01
MS4 + 6-aminouracil→ MS12B1	0.50	0.92	1.31	1.73	2.14	2.56	2.98	3.39	3.81	4.23	4.66
MS4 + 6-aminouracil→ MS13B1 + propanone	3.28	3.32	3.36	3.41	3.45	3.50	3.54	3.59	3.64	3.69	3.75
MS13A1 → TS11A1	3.21	3.20	3.17	3.16	3.14	3.12	3.10	3.09	3.07	3.06	3.04
MS13A1 → MS14A1	-14.71	-14.77	-14.84	-14.91	-14.98	-15.04	-15.12	-15.18	-15.25	-15.32	-15.39
MS13A1 → MS15A1 + CO_2	-19.20	-19.53	-19.87	-20.20	-20.54	-20.87	-21.22	-21.55	-21.88	-22.22	-22.56
MS13A1 → MS16A1 + CO_2	-35.64	-35.96	-36.27	-36.59	-36.90	-37.22	-37.54	-37.85	-38.16	-38.47	-38.78
MS13A2 → TS11A2	5.52	5.56	5.58	5.61	5.64	5.67	5.70	5.73	5.76	5.79	5.83
MS13A2 → MS14A2	-9.83	-9.88	-9.94	-10.00	-10.06	-10.11	-10.18	-10.24	-10.30	-10.36	-10.42
MS13A2 → MS15A2 + CO_2	-14.40	-14.72	-15.05	-15.37	-15.70	-16.03	-16.36	-16.70	-17.02	-17.35	-17.68
MS13A2 → MS16A2 + CO_2	-32.79	-33.09	-33.39	-33.70	-34.00	-34.30	-34.61	-34.92	-35.22	-35.52	-35.82
MS13A1 → TS11B1	4.09	4.07	4.04	4.01	3.99	3.96	3.94	3.91	3.88	3.86	3.83
MS13A1 → MS14B1	-10.57	-10.65	-10.75	-10.84	-10.92	-11.02	-11.11	-11.20	-11.30	-11.39	-11.49

MS13A1 → MS15B1 + CO_2	-15.10	-15.43	-15.77	-16.11	-16.45	-16.79	-17.13	-17.48	-17.81	-18.15	-18.50
MS13A1 → MS16B1 + CO_2	-33.16	-33.47	-33.79	-34.10	-34.42	-34.73	-35.05	-35.37	-35.68	-35.98	-36.31

Table S4: Activation thermodynamic properties (in kcal mol^{-1}) and rate constants (units of L mol and s) for the reaction between benzaldehyde, meldrum's acid and 6-aminouracil in aqueous solution at 298 K and 368 K.

	**Knoevenagel condensation**				
**Step**	**Process**	**ΔG‡(298 K)**	**k (298 K)**	**ΔG‡(368 K)**	**k (368 K)**
1	Meldrum's acid → enol	18.87	9.13×10^{-2}	22.15	5.34×10^{-1}
-1	enol → Meldrum's acid	10.00	2.90×10^{5}	10.00	8.82×10^{6}
2	enol + benzaldehyde → MS5c	19.88	1.66×10^{-2}	22.94	1.82×10^{-1}
-2	MS5c → enol + benzaldehyde	22.64	1.57×10^{-4}	22.65	2.71×10^{-1}
3	MS5c → MS5d	12.61	3.54×10^{3}	13.94	4.03×10^{4}
-3	MS5d → MS5c	10.00	2.90×10^{5}	10.00	8.82×10^{6}
4	MS5d → MS4 + H_2O	17.20	1.53×10^{0}	17.09	5.43×10^{2}
-4	MS4 + H_2O → MS5d	25.81	7.47×10^{-7}	27.92	2.01×10^{-4}
	**Michael addition**				
**Step**	**Process**	**ΔG‡(298 K)**	**k (298 K)**	**ΔG‡(368 K)**	**k (368 K)**
5	MS4 + 6-aminouracil → MS6A	19.03	6.97×10^{-2}	22.35	4.08×10^{-1}
-5	MS6A → MS4 + 6-aminouracil	5.61	4.80×10^{8}	5.69	3.20×10^{9}
6	MS4 + 6-aminouracil → MS6B	17.29	1.31×10^{0}	20.55	4.79×10^{0}
-6	MS6B → MS4 + 6-aminouracil	7.24	3.06×10^{7}	7.21	4.01×10^{8}
7	MS6A + H_2O → MS6A-H_2O	10.26	1.87×10^{5}	12.26	4.01×10^{5}
-7	MS6A-H_2O → MS6A+ H_2O	10.00	2.90×10^{5}	10.00	8.82×10^{6}
8	MS6B + H_2O → MS6B-H_2O	10.20	2.07×10^{5}	12.14	4.73×10^{5}
-8	MS6B-H_2O → MS6B+ H_2O	10.00	2.90×10^{5}	10.00	8.82×10^{6}
9	MS6A-H_2O → MS7A-H_2O	3.83	9.68×10^{9}	4.08	2.89×10^{10}
-9	MS7A-H_2O → MS6A-H_2O	0.40	3.16×10^{12}	0.59	3.42×10^{12}
10	MS6B-H_2O → MS7B-H_2O	5.85	3.20×10^{8}	6.08	1.88×10^{9}
-10	MS7B-H_2O → MS6B-H_2O	-1.64	9.90×10^{13}	-1.49	5.88×10^{13}
11	MS7A-H_2O → MS7A + H_2O	12.79	2.62×10^{3}	10.83	2.84×10^{6}
-11	MS7A + H_2O → MS7A-H_2O	10.00	2.90×10^{5}	10.00	8.82×10^{6}
12	MS7B-H_2O → MS7B + H_2O	8.18	6.27×10^{6}	6.24	1.51×10^{9}
-12	MS7B + H_2O → MS7B-H_2O	10.00	2.90×10^{5}	10.00	8.82×10^{6}
13	MS7A → MS8A	10.64	9.85×10^{4}	5.64	3.43×10^{9}

-13	MS8A → MS7A	20.00	1.36×10^{-2}	15.00	9.46×10^{3}
14	MS7B → MS8B	9.65	5.24×10^{5}	4.60	1.42×10^{10}
-14	MS8B → MS7B	15.00	6.27×10^{1}	11.28	1.54×10^{6}
	**Cyclisation**				
**Step**	**Process**	**ΔG‡(298 K)**	**k (298 K)**	**ΔG‡(368 K)**	**k (368 K)**
15	MS8A → MS10A1	12.97	1.93×10^{3}	13.28	9.94×10^{4}
-15	MS10A1 → MS8A	0.72	1.84×10^{12}	0.96	2.06×10^{12}
16	MS8A → MS10A2	13.58	6.89×10^{2}	13.88	4.38×10^{4}
-16	MS10A2 → MS8A	2.55	8.39×10^{10}	2.68	1.96×10^{11}
17	MS8B → MS10B1	11.52	2.23×10^{4}	11.87	6.84×10^{5}
-17	MS10B1 → MS8B	1.49	5.02×10^{11}	1.61	8.48×10^{11}
18	MS10A1 → MS11A1	6.79	6.54×10^{7}	6.83	6.73×10^{8}
-18	MS11A1 → MS10A1	25.00	2.93×10^{-6}	25.00	1.09×10^{-2}
19	MS10A2 → MS11A2	3.26	2.53×10^{10}	3.12	1.08×10^{11}
-19	MS11A2 → MS10A2	27.00	1.00×10^{-7}	27.00	7.07×10^{4}
20	MS10B1 → MS11B1	2.93	4.42×10^{10}	4.87	9.83×10^{9}
-20	MS11B1 → MS10B1	25.00	2.93×10^{-6}	27.00	7.07×10^{-4}
	**Propanone release**				
**Step**	**Process**	**ΔG‡(298 K)**	**k (298 K)**	**ΔG‡(368 K)**	**k (368 K)**
21	MS11A1 → MS12A1	23.12	7.00×10^{-5}	22.83	2.12×10^{-1}
-21	MS12A1 → MS11A1	12.44	4.72×10^{3}	12.90	1.67×10^{5}
22	MS12A1 → MS13A1 + propanone	7.46	2.11×10^{7}	10.06	8.13×10^{6}
-22	MS13A1 + propanone → MS12A1	15.00	6.27×10^{1}	20.00	1.02×10^{1}
23	MS11A2 → MS12A2	27.44	4.77×10^{-8}	27.21	5.30×10^{-4}
-23	MS12A2 → MS11A2	16.41	5.81×10^{0}	16.90	7.04×10^{2}
24	MS12A2 → MS13A2 + propanone	8.99	1.60×10^{6}	11.58	1.02×10^{6}
-24	MS13A2 + propanone → MS12A2	15.00	6.27×10^{1}	20.00	1.02×10^{1}
25	MS11B1 → MS12B1	26.95	1.09×10^{-7}	26.63	1.17×10^{-3}
-25	MS12B1 → MS11B1	24.98	3.03×10^{-6}	25.25	7.74×10^{-3}
26	MS12B1 → MS13B1 + propanone	12.78	2.66×10^{3}	10.20	6.71×10^{6}
-26	MS13B1 + propanone → MS12B1	10.00	2.90×10^{5}	10.00	8.82×10^{6}
	**CO_2 release**				
**Step**	**Process**	**ΔG‡(298 K)**	**k (298 K)**	**ΔG‡(368 K)**	**k (368 K)**
27	MS13A1 → MS14A1	3.10	3.32×10^{10}	2.20	3.79×10^{11}
-27	MS14A1 → MS13A1	17.91	4.62×10^{-1}	18.26	1.10×10^{2}
28	MS14A1 → MS15A1 + CO_2	5.50	5.77×10^{8}	13.62	6.25×10^{4}
-28	MS15A1 + CO_2 → MS14A1	10.00	2.90×10^{5}	20.00	1.02×10^{1}

29	MS15A1 + CO_2 → MS16A1+ CO_2	3.56	1.53 x 10^{10}	3.70	4.87 x 10^{10}
-29	MS16A1 + CO_2 → MS15A1+ CO_2	20.00	1.36 x 10^{-2}	20.00	1.02 x 10^{1}
30	MS13A2 → MS14A2	5.40	6.84 x 10^{8}	4.83	1.04 x 10^{10}
-30	MS14A2 → MS13A2	15.34	3.53 x 10^{1}	15.96	2.55 x 10^{3}
31	MS14A2 → MS15A2+ CO_2	5.43	6.50 x 10^{8}	13.54	6.97 x 10^{4}
-31	MS15A2 + CO_2 → MS14A2	10.00	2.90 x 10^{5}	20.00	1.02 x 10^{1}
32	MS15A2 + CO_2 → MS16A2 + CO_2	6.61	8.87 x 10^{7}	11.78	7.73 x 10^{5}
-32	MS16A2 + CO_2 → MS15A2+ CO_2	25.00	2.93 x 10^{-6}	30.00	1.17 x 10^{-5}
33	MS13B1 → MS14B1	3.98	7.51 x 10^{9}	3.02	1.23 x 10^{11}
-33	MS14B1 → MS13B1	14.66	1.11 x 10^{2}	15.11	8.14 x 10^{3}
34	MS14B1 → MS15B1 + CO_2	5.46	6.18 x 10^{8}	13.71	5.52 x 10^{4}
-34	MS15B1 + CO_2 → MS14B1	10.00	2.90 x 10^{5}	20.00	1.02 x 10^{1}
35	MS15B1 + CO_2 → MS16B1+ CO_2	6.94	5.08 x 10^{7}	12.11	4.93 x 10^{5}
-35	MS16B1 + CO_2 → MS15B1+ CO_2	25.00	2.93 x 10^{-6}	30.00	1.17 x 10^{-5}

Microkinetic model

$$\text{Meldrum's acid} \underset{k_{-1}}{\overset{k_1}{\rightleftharpoons}} enol \quad \text{(S1)}$$

$$\text{Enol + benzaldehyde} \underset{k_{-2}}{\overset{k_2}{\rightleftharpoons}} \text{MS5c} \quad \text{(S2)}$$

$$\text{MS5c} \underset{k_{-3}}{\overset{k_3}{\rightleftharpoons}} \text{MS5d} \quad \text{(S3)}$$

$$\text{MS5d} \underset{k_{-4}}{\overset{k_4}{\rightleftharpoons}} \text{MS4} + H_2O \quad \text{(S4)}$$

$$\text{MS4 + 6-aminouracil} \underset{k_{-5}}{\overset{k_5}{\rightleftharpoons}} \text{MS6A} \quad \text{(S5)}$$

$$\text{MS4 + 6-aminouracil} \underset{k_{-6}}{\overset{k_6}{\rightleftharpoons}} \text{MS6B} \quad \text{(S6)}$$

$$MS6A + H_2O \underset{k_{-7}}{\overset{k_7}{\rightleftharpoons}} MS6A\text{-}H_2O \tag{S7}$$

$$MS6B + H_2O \underset{k_{-8}}{\overset{k_8}{\rightleftharpoons}} MS6B\text{-}H_2O$$

(S8)

$$MS6A\text{-}H_2O \underset{k_{-9}}{\overset{k_9}{\rightleftharpoons}} MS7A\text{-}H_2O \tag{S9}$$

$$MS6B\text{-}H_2O \underset{k_{-10}}{\overset{k_{10}}{\rightleftharpoons}} MS7B\text{-}H_2O \tag{S10}$$

$$MS7A\text{-}H_2O \underset{k_{-11}}{\overset{k_{11}}{\rightleftharpoons}} MS7A + H_2O \tag{S11}$$

$$MS7B\text{-}H_2O \underset{k_{-12}}{\overset{k_{12}}{\rightleftharpoons}} MS7B + H_2O \tag{S12}$$

$$MS7A \underset{k_{-13}}{\overset{k_{13}}{\rightleftharpoons}} MS8A \tag{S13}$$

$$MS7B \underset{k_{-14}}{\overset{k_{14}}{\rightleftharpoons}} MS8B \tag{S14}$$

$$MS8A \underset{k_{-15}}{\overset{k_{15}}{\rightleftharpoons}} MS10A1 \tag{S15}$$

$$MS8A \underset{k_{-16}}{\overset{k_{16}}{\rightleftharpoons}} MS10A2 \tag{S16}$$

$$\mathrm{MS8B} \underset{k_{-17}}{\overset{k_{17}}{\rightleftharpoons}} \mathrm{MS10B1} \tag{S17}$$

$$\mathrm{MS10A1} \underset{k_{-18}}{\overset{k_{18}}{\rightleftharpoons}} \mathrm{MS11A1} \tag{S18}$$

$$\mathrm{MS10A2} \underset{k_{-19}}{\overset{k_{19}}{\rightleftharpoons}} \mathrm{MS11A2} \tag{S19}$$

$$\mathrm{MS10B1} \underset{k_{-20}}{\overset{k_{20}}{\rightleftharpoons}} \mathrm{MS11B1} \tag{S20}$$

$$\mathrm{MS11A1} \underset{k_{-21}}{\overset{k_{21}}{\rightleftharpoons}} \mathrm{MS12A1} \tag{S21}$$

$$\mathrm{MS12A1} \underset{k_{-22}}{\overset{k_{22}}{\rightleftharpoons}} \mathrm{MS13A1} + \text{propanone} \tag{S22}$$

$$\mathrm{MS11A2} \underset{k_{-23}}{\overset{k_{23}}{\rightleftharpoons}} \mathrm{MS12A2} \tag{S23}$$

$$\mathrm{MS12A2} \underset{k_{-24}}{\overset{k_{24}}{\rightleftharpoons}} \mathrm{MS13A2} + \text{propanone} \tag{S24}$$

$$\mathrm{MS11B1} \underset{k_{-25}}{\overset{k_{25}}{\rightleftharpoons}} \mathrm{MS12B1} \tag{S25}$$

$$\mathrm{MS12B1} \underset{k_{-26}}{\overset{k_{26}}{\rightleftharpoons}} \mathrm{MS3B1} + \text{propanone} \tag{S26}$$

$$\mathrm{MS13A1} \underset{k_{-27}}{\overset{k_{27}}{\rightleftharpoons}} \mathrm{MS14A1} \tag{S27}$$

$$\mathrm{MS14A1} \underset{k_{-28}}{\overset{k_{28}}{\rightleftharpoons}} \mathrm{MS15A1} + CO_2 \quad \text{(S28)}$$

$$\mathrm{MS5A1} + CO_2 \underset{k_{-29}}{\overset{k_{29}}{\rightleftharpoons}} \mathrm{MS16A1} + CO_2 \quad \text{(S29)}$$

$$\mathrm{MS13A2} \underset{k_{-30}}{\overset{k_{30}}{\rightleftharpoons}} \mathrm{MS14A2} \quad \text{(S30)}$$

$$\mathrm{MS14A2} \underset{k_{-31}}{\overset{k_{31}}{\rightleftharpoons}} \mathrm{MS15A2} + CO_2 \quad \text{(S31)}$$

$$\mathrm{MS15A2} + CO_2 \underset{k_{-32}}{\overset{k_{32}}{\rightleftharpoons}} \mathrm{MS16A2} + CO_2 \quad \text{(S32)}$$

$$\mathrm{MS13B1} \underset{k_{-33}}{\overset{k_{33}}{\rightleftharpoons}} \mathrm{MS14B1} \quad \text{(S33)}$$

$$\mathrm{MS14B1} \underset{k_{-34}}{\overset{k_{34}}{\rightleftharpoons}} \mathrm{MS15B1} + CO_2 \quad \text{(S34)}$$

$$\mathrm{MS15B1} + CO_2 \underset{k_{-35}}{\overset{k_{35}}{\rightleftharpoons}} \mathrm{MS16B1} + CO_2 \quad \text{(S35)}$$

Coordinates of the optimized structures.

Coordinates of the optimized structures, obtained with the X3LYP functional and the def2-SVP basis set. Solvent effect obtained with the continuum SMD method in water.

Benzaldehyde

C	-0.261372	2.556018	-0.757337
O	0.065874	1.424622	-1.074059

H	-1.326299	2.792640	-0.521965
C	0.662978	3.699943	-0.650890
C	0.159450	4.958369	-0.283755
C	2.036209	3.541829	-0.913927
C	1.019972	6.052607	-0.180592
H	-0.909463	5.071298	-0.081507
C	2.892434	4.634644	-0.809496
H	2.412595	2.556486	-1.197920
C	2.384197	5.889533	-0.443446
H	0.629561	7.032350	0.103965
H	3.959548	4.516451	-1.012069
H	3.059087	6.745439	-0.363433

6-Aminouracil

C	-5.611217	1.156414	-0.192380
C	-4.420794	1.910995	0.013127
N	-3.256257	1.140508	0.210742
O	-4.322549	3.144535	0.034186
C	-5.577776	-0.229066	-0.189903
H	-6.550146	1.684308	-0.350099
N	-6.648872	-1.012895	-0.385259
H	-7.564486	-0.591947	-0.487885
H	-6.581694	-2.021970	-0.307363
N	-4.375483	-0.872223	0.015417
C	-3.173549	-0.225785	0.223946
H	-4.336760	-1.890391	0.019393
H	-2.386025	1.648613	0.358484
O	-2.129183	-0.841316	0.404515

Meldrum's acid

C	-3.417367	1.453007	-0.367155
C	-2.206142	0.942907	0.367596
C	-0.897539	1.488282	-0.140331
H	-2.187735	-0.153522	0.334090
H	-2.306050	1.231641	1.429803
O	-3.303337	2.672270	-0.928650
O	-0.946668	2.699045	-0.729417
C	-2.144061	3.503234	-0.711382
C	-2.262916	4.240102	0.611689
C	-2.055148	4.420123	-1.910630
O	-4.447788	0.837007	-0.487051
O	0.155714	0.904508	-0.066737
H	-2.332670	3.546352	1.461681
H	-3.161923	4.871990	0.599119
H	-1.380438	4.879848	0.752488
H	-1.973433	3.823425	-2.830253
H	-1.172799	5.068176	-1.818529
H	-2.955629	5.047368	-1.963771

Enol

C	-3.383040	1.445166	-0.401954
C	-2.139754	0.879434	0.039945
C	-0.974641	1.514005	-0.285826
H	-2.128200	-0.097925	0.517340
O	-3.294945	2.678441	-1.020609
O	-0.945551	2.712221	-0.863875
C	-2.163218	3.510356	-0.781866
C	-2.243051	4.155481	0.592787
C	-2.087596	4.503524	-1.918381
O	-4.484712	0.928165	-0.340610

O	0.215157	0.977882	-0.084868
H	-2.273497	3.400348	1.391312
H	-3.156115	4.764770	0.651601
H	-1.371252	4.806030	0.750309
H	-2.023876	3.968555	-2.876682
H	-1.202549	5.143777	-1.799318
H	-2.986795	5.134968	-1.916263
H	0.914866	1.613831	-0.322870

Water

O	-8.286660	0.324068	-0.013507
H	-7.319707	0.375673	0.018005
H	-8.551973	1.119349	0.472092

Meldrum's acid anion

C	-3.404104	1.536063	-0.197167
C	-2.204359	1.027722	0.345343
C	-0.942389	1.566902	0.016003
H	-2.243064	0.104200	0.922590
O	-3.294395	2.707858	-0.943065
O	-0.952681	2.735650	-0.743245
C	-2.141161	3.529857	-0.748855
C	-2.261915	4.309435	0.558798
C	-2.048648	4.437055	-1.961043
O	-4.537251	1.052349	-0.125661
O	0.173854	1.114216	0.284447
H	-2.334560	3.630433	1.420195
H	-3.160056	4.943642	0.534223
H	-1.379978	4.952860	0.691984
H	-1.960293	3.827527	-2.872305

H	-1.168036	5.090142	-1.879673
H	-2.949145	5.063388	-2.034100

Protonated 6-aminouracil

C	-5.614199	1.168221	-0.164651
C	-4.403956	1.930776	0.115300
N	-3.254260	1.150826	0.253594
O	-4.351340	3.147081	0.226782
C	-5.522183	-0.172340	-0.261368
H	-6.560009	1.693390	-0.289821
N	-6.685081	-0.995340	-0.539519
H	-7.529137	-0.412571	-0.659399
H	-6.874327	-1.665524	0.228893
N	-4.353450	-0.859100	-0.109175
C	-3.150286	-0.220155	0.157426
H	-4.319570	-1.876358	-0.192034
H	-2.384058	1.648223	0.446455
O	-2.105575	-0.829716	0.290999
H	-6.563700	-1.555364	-1.403591

PhOH

H	-3.022759	-3.272239	3.875851
C	-3.649010	-2.909298	3.057499
C	-4.995068	-3.301293	3.005318
C	-5.815104	-2.846135	1.962784
H	-6.863348	-3.157072	1.930427
C	-5.287161	-2.002849	0.981378
H	-5.934686	-1.652999	0.173074
C	-3.946467	-1.607401	1.027767
H	-3.538147	-0.947930	0.258646

C	-3.133671	-2.066255	2.071178
H	-2.084089	-1.764426	2.120062
O	-5.459672	-4.124361	3.990191
H	-6.400147	-4.309744	3.841594

PhO^-

H	-3.009002	-3.260878	3.863789
C	-3.648062	-2.902926	3.048805
C	-5.019437	-3.342079	3.048418
C	-5.807474	-2.838640	1.953357
H	-6.858764	-3.144824	1.911234
C	-5.278502	-1.997126	0.976413
H	-5.927151	-1.645665	0.166233
C	-3.934504	-1.592638	1.012491
H	-3.524786	-0.932812	0.243680
C	-3.132282	-2.060672	2.065519
H	-2.079924	-1.759869	2.118023
O	-5.499993	-4.119381	3.951548

$PhNH_2$

H	-3.032156	-3.217431	3.894230
C	-3.666128	-2.872045	3.072369
C	-5.024793	-3.254741	3.047274
C	-5.822398	-2.802845	1.973451
H	-6.876262	-3.093405	1.935684
C	-5.279350	-1.996864	0.972328
H	-5.920282	-1.662645	0.151530
C	-3.932216	-1.617597	1.006373
H	-3.511347	-0.987159	0.219834
C	-3.134180	-2.065039	2.066015

H	-2.078033	-1.784494	2.110911
N	-5.571666	-4.007589	4.071799
H	-6.418987	-4.517269	3.839229
H	-4.913502	-4.563278	4.610075

$PhNH_3^+$

H	4.257400	1.577795	-0.931149
C	3.700656	2.489400	-1.158471
C	2.311454	2.523361	-1.007348
H	1.779552	1.634128	-0.661618
C	1.601215	3.693723	-1.293721
H	0.516279	3.723124	-1.172376
C	2.275445	4.835418	-1.732869
H	1.734048	5.757539	-1.957009
C	3.660076	4.778597	-1.877843
C	4.387333	3.624967	-1.597103
H	5.473280	3.616064	-1.716786
N	4.373339	5.971234	-2.353436
H	3.996100	6.835246	-1.935135
H	5.378562	5.929553	-2.129866
H	4.295721	6.079631	-3.377578

CH_3NH_2

C	-8.979392	3.009835	-0.300960
N	-8.027263	2.386647	0.615350
H	-7.261690	3.044143	0.779138
H	-8.475993	2.291091	1.528991
H	-9.386012	3.986683	0.029509
H	-9.839712	2.341767	-0.466831
H	-8.508679	3.172325	-1.283835

$CH_3NH_3^+$

C	-9.008606	3.017366	-0.339683
N	-8.047340	2.427789	0.628892
H	-7.232329	3.040372	0.772397
H	-8.483244	2.276643	1.549006
H	-7.693985	1.517505	0.302229
H	-9.366374	3.974503	0.056753
H	-9.848549	2.325402	-0.469702
H	-8.495244	3.172388	-1.295642

H_3O^+

O	1.822644	2.048777	-1.006845
H	2.784575	2.234371	-0.926858
H	1.495348	1.791620	-0.116898
H	1.376404	2.888271	-1.256349

TS1k

C	-2.545423	0.351723	-0.234360
C	-3.217570	0.886568	-1.424892
C	-4.562472	1.442672	-1.219186
H	-2.586904	1.623114	-1.929507
O	-3.315481	0.024999	0.834866
O	-5.221693	1.123801	-0.076791
C	-4.749070	0.142161	0.860598
C	-5.111212	0.658784	2.239432
C	-5.366409	-1.203441	0.527471
O	-1.346645	0.160604	-0.138501
O	-5.128994	2.172324	-2.012856
H	-4.634643	1.634808	2.411773

H	-4.768035	-0.050950	3.005158
H	-6.201590	0.769867	2.321515
H	-5.060700	-1.501621	-0.486603
H	-6.462605	-1.131263	0.582241
H	-5.024522	-1.956804	1.252399
H	0.332957	1.145061	-5.250523
C	-0.071598	0.361862	-4.603687
H	-1.959811	1.372449	-4.319536
C	-1.360296	0.488267	-4.079027
C	0.698459	-0.769700	-4.307851
H	1.706076	-0.871799	-4.719234
C	-1.900716	-0.508606	-3.252047
C	0.165660	-1.771764	-3.490680
H	-3.921683	0.230182	-3.415556
C	-3.329624	-0.407534	-2.709994
C	-1.126321	-1.641579	-2.971356
H	0.758016	-2.661919	-3.261249
O	-3.876720	-1.505059	-2.296231
H	-1.558593	-2.427097	-2.348067

MS5a

C	-3.499330	1.821668	0.081215
C	-2.253119	1.020781	0.251850
C	-1.069920	1.507248	-0.513274
O	-3.417262	3.025715	-0.524286
O	-1.100075	2.763444	-1.007262
C	-2.176827	3.696843	-0.799759
C	-1.834455	4.602844	0.368601
C	-2.368817	4.426401	-2.113623
O	-4.592661	1.435570	0.439578

O	-0.077033	0.841530	-0.717918
H	-1.713137	3.993341	1.275004
H	-2.639985	5.336816	0.515679
H	-0.897910	5.140064	0.159483
H	-2.615974	3.707118	-2.907808
H	-1.445577	4.958750	-2.381583
H	-3.185422	5.155833	-2.018945
H	-2.486225	0.001724	-0.081988
H	-2.673866	-1.727700	1.471991
H	-2.866995	0.690383	2.293713
C	-1.635886	-1.665633	1.815808
H	-1.379691	-3.811046	1.848413
C	-1.850592	0.905268	1.863805
C	-0.910219	-2.840044	2.029081
C	-1.054778	-0.407341	2.042487
O	-1.249435	1.994436	2.321837
C	0.415237	-2.775725	2.478134
C	0.266142	-0.356264	2.502411
H	0.984171	-3.693808	2.646991
C	1.000146	-1.528666	2.715561
H	0.701478	0.625676	2.698121
H	2.032378	-1.468154	3.072185

MS5b

C	-3.035749	1.860867	0.668858
C	-1.709439	1.849165	1.160127
C	-0.651175	2.381768	0.393987
O	-3.251266	2.538552	-0.524294
O	-0.967334	3.026262	-0.784573

C	-2.303138	3.523646	-0.937744
C	-2.480159	4.803164	-0.125516
C	-2.515601	3.736138	-2.423526
O	-4.025052	1.300453	1.152383
O	0.558631	2.315386	0.674536
H	-2.311438	4.620226	0.945340
H	-3.500795	5.189779	-0.259597
H	-1.765834	5.564948	-0.469824
H	-2.378218	2.784346	-2.957095
H	-1.792748	4.470298	-2.806802
H	-3.532848	4.108649	-2.609970
H	-2.797482	-0.978474	1.476592
H	-2.280912	1.059075	3.051149
C	-1.764102	-1.265900	1.686448
H	-2.050786	-3.305132	1.039060
C	-1.359577	1.126683	2.450829
C	-1.342862	-2.573851	1.438558
C	-0.874663	-0.307965	2.204640
C	-0.019918	-2.950188	1.701713
C	0.443682	-0.697320	2.474328
H	0.311451	-3.973340	1.506484
C	0.870199	-2.006650	2.219690
H	1.141102	0.030739	2.891840
H	1.904820	-2.289274	2.432619
H	0.260610	2.144706	2.582317
O	-0.425890	1.874424	3.222811

MS5d

C	-2.769347	1.644911	0.353965
C	-1.640603	1.908351	1.207312

C	-0.666009	2.760290	0.742852
O	-2.839616	2.366778	-0.814814
O	-0.787764	3.436726	-0.397954
C	-2.128474	3.599726	-0.927303
C	-2.841386	4.704348	-0.161831
C	-1.968292	3.890589	-2.402050
O	-3.629496	0.800346	0.547453
O	0.486655	2.996529	1.332998
H	-2.920367	4.466906	0.908725
H	-3.854404	4.830572	-0.569401
H	-2.292203	5.649362	-0.277327
H	-1.447464	3.056087	-2.892663
H	-1.387766	4.813290	-2.539954
H	-2.957664	4.020524	-2.861850
H	-2.889853	-1.216777	2.911626
H	-2.470662	1.055470	2.981129
C	-1.895270	-1.414674	2.503311
H	-2.159940	-3.560175	2.492288
C	-1.490062	1.091093	2.478230
C	-1.484519	-2.731158	2.266375
C	-1.042242	-0.341165	2.218323
C	-0.212022	-2.984601	1.748096
C	0.234850	-0.603223	1.694466
H	0.111910	-4.012317	1.565409
C	0.647727	-1.916518	1.463799
H	0.906603	0.228125	1.465724
H	1.643951	-2.108923	1.057391
H	0.441538	2.537982	2.220736
O	-0.552469	1.782126	3.322655
H	-0.206909	1.160750	3.982432

TS2k

C	-3.143746	1.891190	0.034784
C	-1.925401	1.624486	0.748975
C	-0.727023	2.341159	0.448558
O	-3.049742	2.791842	-0.999205
O	-0.764011	3.223851	-0.589890
C	-2.029082	3.793777	-0.975520
C	-2.405811	4.895214	0.007013
C	-1.865709	4.284740	-2.397717
O	-4.225547	1.343815	0.203390
O	0.328808	2.254843	1.084530
H	-2.501583	4.499626	1.029438
H	-3.366044	5.341497	-0.288547
H	-1.633674	5.677753	0.003424
H	-1.596133	3.443114	-3.052010
H	-1.074259	5.045897	-2.442989
H	-2.807111	4.728756	-2.750444
H	-2.275484	-1.211890	3.684633
H	-2.981532	0.536180	2.224841
C	-1.457146	-1.446036	2.998088
H	-0.999519	-3.327851	3.949325
C	-1.967631	0.688458	1.842263
C	-0.742665	-2.633852	3.145731
C	-1.138698	-0.536035	1.970397
C	0.292401	-2.938630	2.255517
C	-0.104135	-0.859128	1.074204
H	0.850069	-3.872184	2.363214
C	0.604475	-2.052233	1.219002
H	0.140906	-0.184320	0.254289

H	1.402996	-2.294926	0.514019
H	-0.492346	2.005752	2.824892
O	-1.337840	1.714040	3.245756
H	-1.069317	1.142447	3.990199

MS4

C	-0.136646	2.698216	-0.191242
H	-1.068341	2.674405	0.383536
C	-0.014435	3.896863	-1.005650
C	0.871604	4.072148	-2.093652
C	-0.915123	4.945004	-0.698215
C	0.862517	5.257265	-2.823572
H	1.557092	3.275276	-2.372050
C	-0.902882	6.138339	-1.415979
H	-1.625304	4.809214	0.121025
C	-0.012678	6.296672	-2.482581
H	1.544406	5.375342	-3.668885
H	-1.596057	6.940439	-1.153084
H	-0.005662	7.226650	-3.056392
H	0.258319	-0.502081	-1.768192
C	0.857916	-1.350501	-1.406746
H	0.190652	-2.206471	-1.233913
H	1.589610	-1.619867	-2.181265
O	0.647183	-0.660349	0.911763
O	2.484712	0.095122	-0.330785
C	1.587753	-1.006713	-0.117880
C	0.027792	0.540411	0.892546
C	2.017893	1.361313	-0.440469
O	-0.942464	0.702907	1.601305
O	2.789203	2.219001	-0.812112

C	0.625331	1.580088	0.009829
C	2.424490	-2.141398	0.429543
H	1.782667	-3.007902	0.639915
H	3.184863	-2.431063	-0.308703
H	2.921338	-1.823817	1.357284

TS3k

C	-3.008638	1.618177	0.325985
C	-1.660421	1.158268	0.166920
C	-0.756120	1.836717	-0.698941
H	-1.285066	0.397395	0.846434
O	-3.341587	2.749056	-0.390280
O	-1.175707	2.985714	-1.283880
C	-2.307177	3.681231	-0.715445
C	-1.865251	4.447043	0.524604
C	-2.850273	4.574273	-1.809687
O	-3.899574	1.063120	0.951693
O	0.349394	1.398126	-1.069198
H	-1.460616	3.771113	1.292142
H	-2.723547	4.987574	0.948723
H	-1.087657	5.174648	0.252096
H	-3.148117	3.964366	-2.674547
H	-2.081181	5.294024	-2.122442
H	-3.725297	5.126217	-1.439141
H	0.085307	-0.155691	-1.356653
H	-1.998654	0.074546	-2.139724
O	-0.475740	-1.000180	-1.258683
C	-1.726491	-0.685869	-1.399463
H	-4.336840	-0.424357	-1.855474
C	-2.756848	-1.575307	-0.917346

C	-4.100070	-1.303624	-1.251752
C	-2.440817	-2.707177	-0.134884
C	-5.110726	-2.158441	-0.817557
H	-1.399966	-2.911618	0.121672
H	-6.150845	-1.951738	-1.078268
C	-3.456483	-3.553998	0.294112
C	-4.789710	-3.280929	-0.046426
H	-3.216098	-4.433264	0.895751
H	-5.583364	-3.950845	0.293308

MS5c

C	-3.497254	2.097946	0.461601
C	-2.296673	1.169882	0.472924
C	-1.150438	1.567712	-0.435753
O	-3.466820	3.157271	-0.363113
O	-1.287008	2.684907	-1.168497
C	-2.237055	3.720652	-0.853959
C	-1.647476	4.637501	0.203570
C	-2.569485	4.406815	-2.159427
O	-4.497750	1.872826	1.099152
O	-0.159683	0.891057	-0.575749
H	-1.444752	4.072894	1.125639
H	-2.356351	5.446937	0.427464
H	-0.709430	5.074297	-0.166667
H	-2.990824	3.677913	-2.866398
H	-1.658421	4.845497	-2.589165
H	-3.301496	5.206654	-1.981860
H	-2.489243	-1.700015	1.145240
H	-2.798553	0.659221	2.472670
C	-1.499093	-1.643809	1.606403

H	-1.149442	-3.761198	1.390269
C	-1.857015	0.848688	1.935322
C	-0.745217	-2.810130	1.746062
C	-1.002612	-0.409499	2.057347
C	0.520616	-2.763709	2.341673
C	0.258088	-0.374847	2.666547
H	1.112078	-3.676225	2.449707
C	1.016965	-1.542997	2.802618
H	0.655080	0.567276	3.048787
H	2.000662	-1.494100	3.276582
H	-0.434426	2.187090	2.152314
O	-1.291367	1.981265	2.559353
H	-2.686364	0.233557	0.037355

TS4kA

C	-0.103977	2.576048	-0.378665
H	-0.832144	2.648833	0.431868
C	0.313986	3.940139	-0.813315
C	1.048795	4.233743	-1.978328
C	-0.127306	5.017255	-0.017675
C	1.330643	5.555408	-2.327458
H	1.405584	3.425106	-2.611515
C	0.173168	6.338154	-0.357029
H	-0.710452	4.811908	0.883482
C	0.900630	6.612215	-1.517703
H	1.895590	5.761412	-3.239954
H	-0.171015	7.153758	0.283600
H	1.128704	7.644741	-1.793752
H	0.713566	-1.141128	-1.639421
C	1.385490	-1.795505	-1.066789

H	0.825753	-2.684878	-0.743301
H	2.208366	-2.115933	-1.722267
O	0.886270	-0.678436	1.021310
O	2.703506	0.068761	-0.254006
C	1.952409	-1.075269	0.150079
C	0.145924	0.429317	0.720234
C	2.070085	1.227634	-0.652955
O	-0.956362	0.514823	1.254545
O	2.749993	1.994123	-1.320030
C	0.726663	1.420213	-0.163586
C	2.884259	-1.939959	0.974070
H	2.350079	-2.831526	1.331482
H	3.738845	-2.259314	0.361212
H	3.253115	-1.368588	1.838155
H	-3.391775	2.727969	0.102606
N	-3.362148	3.349991	-0.701212
H	-1.904258	1.174009	-1.019719
H	-3.978819	4.160073	-0.715377
C	-1.591527	2.014352	-1.641303
C	-2.515479	3.110225	-1.684228
C	-0.944840	1.641673	-2.895272
O	-0.318310	0.602461	-3.067749
N	-2.471907	3.968125	-2.734906
H	-3.046799	4.812137	-2.734849
N	-1.014949	2.588171	-3.916932
C	-1.716931	3.767294	-3.889079
O	-1.715794	4.578085	-4.795521
H	-0.492510	2.393840	-4.771397

MS6A

C	-0.434315	2.506625	-0.659372
H	-1.070023	2.555245	0.232614
C	0.157539	3.909191	-0.797207
C	0.709786	4.448944	-1.970246
C	0.160462	4.712006	0.358498
C	1.240449	5.743042	-1.986540
H	0.745167	3.858266	-2.885030
C	0.699881	6.000376	0.349631
H	-0.268424	4.315449	1.283106
C	1.241531	6.523849	-0.827876
H	1.661008	6.138783	-2.914590
H	0.688547	6.598674	1.264285
H	1.659284	7.533627	-0.842973
H	0.423553	-1.328697	-1.257372
C	1.233362	-1.846573	-0.724838
H	0.800483	-2.662044	-0.127373
H	1.918192	-2.279947	-1.468377
O	1.127108	-0.363146	1.188836
O	2.573160	0.170407	-0.572527
C	1.999280	-0.891806	0.186840
C	0.236428	0.632006	0.834438
C	1.746588	1.186094	-1.038844
O	-0.727830	0.779934	1.592207
O	2.179168	1.813493	-2.006754
C	0.539445	1.404642	-0.324115
C	3.139446	-1.572073	0.918951
H	2.753238	-2.392931	1.539693
H	3.859232	-1.982337	0.196504
H	3.652501	-0.844342	1.564346
H	-3.288945	3.065624	-0.135893

N	-3.313365	3.544273	-1.035159
H	-2.053616	1.247211	-1.304549
H	-3.974579	4.312701	-1.157685
C	-1.518020	2.081999	-1.779785
C	-2.506767	3.182714	-1.995574
C	-0.957905	1.517530	-3.067383
O	-0.425693	0.428072	-3.126274
N	-2.542196	3.825080	-3.174656
H	-3.171110	4.622851	-3.301824
N	-1.087271	2.300886	-4.203100
C	-1.839301	3.445295	-4.328873
O	-1.934550	4.081966	-5.352634
H	-0.658425	1.951043	-5.061935

TS4kB

C	-0.450011	2.318618	-0.779244
H	0.096794	2.943872	-1.489836
C	-0.716220	3.074041	0.474186
C	-1.293341	2.537397	1.642669
C	-0.322027	4.428486	0.490184
C	-1.453319	3.327434	2.782245
H	-1.620975	1.499796	1.651111
C	-0.477804	5.214708	1.633100
H	0.121998	4.865665	-0.407965
C	-1.045348	4.665818	2.785684
H	-1.901283	2.891252	3.678688
H	-0.155510	6.258704	1.621071
H	-1.171962	5.277461	3.682419
H	2.056406	-0.126562	0.588172
C	2.210342	-1.150667	0.219779

H	3.243811	-1.243094	-0.143553
H	2.065304	-1.851474	1.054280
O	1.427997	-0.600576	-2.013905
O	-0.113613	-1.395666	-0.449021
C	1.240516	-1.488295	-0.906127
C	0.953435	0.680674	-1.932433
C	-0.687571	-0.163952	-0.280648
O	1.377160	1.478873	-2.759411
O	-1.764165	-0.145058	0.308107
C	-0.019533	0.955818	-0.893382
C	1.418019	-2.892905	-1.444147
H	2.436017	-3.017220	-1.839176
H	1.255942	-3.624984	-0.640689
H	0.692709	-3.074850	-2.250262
H	-3.513075	3.796009	0.057949
N	-3.343358	4.331646	-0.789057
H	-2.789467	1.750456	-1.059131
H	-3.581399	5.321260	-0.796622
C	-2.285138	2.402765	-1.773090
C	-2.713922	3.764513	-1.802060
C	-1.968897	1.783280	-3.053523
O	-1.843960	0.575347	-3.220167
N	-2.424548	4.522376	-2.895659
H	-2.647472	5.518937	-2.896940
N	-1.733328	2.663877	-4.110945
C	-1.928631	4.021425	-4.095863
O	-1.711705	4.747424	-5.048925
H	-1.422767	2.261430	-4.995145

MS6B

C	-0.708566	2.351476	-1.073922
H	-0.069414	2.945963	-1.739254
C	-0.719593	3.081009	0.265578
C	-1.295189	2.539778	1.429477
C	-0.119287	4.348373	0.346329
C	-1.265682	3.248472	2.633640
H	-1.766751	1.557382	1.388383
C	-0.086179	5.056256	1.552030
H	0.332281	4.785110	-0.548730
C	-0.661350	4.508500	2.701099
H	-1.717754	2.810737	3.527510
H	0.391677	6.038544	1.590793
H	-0.638515	5.058836	3.645028
H	1.668108	-0.046024	0.866530
C	1.932038	-1.059276	0.532566
H	3.021787	-1.112013	0.395232
H	1.640980	-1.774604	1.315348
O	1.615823	-0.512014	-1.812984
O	-0.187028	-1.383128	-0.610087
C	1.231515	-1.415914	-0.775490
C	1.068419	0.756939	-1.827272
C	-0.817716	-0.153578	-0.543257
O	1.646513	1.579309	-2.542632
O	-1.966558	-0.176109	-0.084033
C	-0.114725	0.964413	-1.060001
C	1.579797	-2.808991	-1.262073
H	2.663219	-2.890194	-1.428911
H	1.279075	-3.555355	-0.513221
H	1.054310	-3.014918	-2.205976
H	-3.596936	3.472466	0.096538

N	-3.486218	4.091396	-0.705194
H	-2.787082	1.638712	-1.225276
H	-3.892288	5.026852	-0.655396
C	-2.166496	2.352985	-1.781362
C	-2.795257	3.701857	-1.741124
C	-1.995692	1.844941	-3.195145
O	-1.913197	0.666245	-3.471053
N	-2.608210	4.538251	-2.778796
H	-2.954448	5.499947	-2.730178
N	-1.846544	2.817652	-4.172060
C	-2.112748	4.159026	-4.034942
O	-1.982857	4.968683	-4.925127
H	-1.602512	2.504328	-5.113244

MS6A-H_2O

C	-0.395354	2.512432	-0.675830
H	-1.037029	2.596994	0.205553
C	0.204076	3.909356	-0.833995
C	0.757437	4.431663	-2.014132
C	0.197055	4.732573	0.306968
C	1.276279	5.729805	-2.052409
H	0.799576	3.826398	-2.919056
C	0.725701	6.025304	0.277042
H	-0.233138	4.350470	1.237088
C	1.265869	6.532008	-0.908134
H	1.696149	6.112913	-2.986038
H	0.705399	6.640047	1.180579
H	1.673207	7.545615	-0.940825
H	0.548881	-1.310980	-1.388510
C	1.320619	-1.842366	-0.813854

H	0.852214	-2.685726	-0.286093
H	2.068120	-2.238067	-1.516960
O	1.043739	-0.439258	1.142064
O	2.614625	0.179728	-0.479683
C	2.000738	-0.917735	0.191322
C	0.178625	0.560176	0.761398
C	1.816135	1.209634	-0.960155
O	-0.855324	0.640742	1.448528
O	2.307889	1.875001	-1.869831
C	0.560429	1.399194	-0.316944
C	3.089440	-1.612171	0.984962
H	2.667704	-2.460921	1.541584
H	3.869007	-1.985429	0.305918
H	3.540005	-0.903742	1.695165
H	-3.272590	2.896336	-0.057213
N	-3.323948	3.420474	-0.972893
H	-1.965830	1.202893	-1.332804
H	-4.017506	4.161996	-1.083333
C	-1.474490	2.075027	-1.787747
C	-2.518734	3.137308	-1.953290
C	-0.900635	1.578053	-3.096645
O	-0.291120	0.531246	-3.190321
N	-2.591783	3.816075	-3.117132
H	-3.252309	4.591189	-3.212535
N	-1.104012	2.373701	-4.210849
C	-1.903362	3.491623	-4.290402
O	-2.042390	4.144801	-5.300229
H	-0.675489	2.067991	-5.086267
O	-3.143256	2.081568	1.400942
H	-2.320849	1.529110	1.379605

H	-2.926505	2.777269	2.041789

MS6B-H_2O

C	-0.660908	2.312713	-1.151394
H	0.035696	2.872560	-1.789763
C	-0.705639	3.083987	0.166257
C	-0.956587	2.474665	1.406020
C	-0.441536	4.464727	0.147609
C	-0.963877	3.227174	2.584600
H	-1.147879	1.403657	1.458177
C	-0.450799	5.219844	1.323687
H	-0.216782	4.954197	-0.804151
C	-0.716625	4.602863	2.549117
H	-1.160335	2.731080	3.538627
H	-0.240380	6.291575	1.282089
H	-0.721401	5.188176	3.471980
H	1.509165	-0.160006	0.951665
C	1.752119	-1.185065	0.637914
H	2.844906	-1.280375	0.562348
H	1.389762	-1.883435	1.406035
O	1.593500	-0.643802	-1.724562
O	-0.310176	-1.428861	-0.620482
C	1.113790	-1.523499	-0.705949
C	1.098985	0.643339	-1.779911
C	-0.884128	-0.175906	-0.590719
O	1.748417	1.437428	-2.461979
O	-2.055247	-0.154566	-0.167810
C	-0.123375	0.905065	-1.088160
C	1.428566	-2.933159	-1.164873

H	2.515347	-3.060146	-1.269138
H	1.054999	-3.660419	-0.430253
H	0.949447	-3.123261	-2.136270
H	-3.804054	2.871358	-0.096741
N	-3.670568	3.684697	-0.758609
H	-2.655679	1.480028	-1.594195
H	-4.191838	4.550043	-0.609741
C	-2.065970	2.326733	-1.962418
C	-2.856506	3.575940	-1.764422
C	-1.730071	2.082211	-3.415153
O	-1.487980	0.980037	-3.863506
N	-2.687922	4.591617	-2.638063
H	-3.140950	5.492458	-2.468622
N	-1.620186	3.216122	-4.204127
C	-2.046768	4.483232	-3.876653
O	-1.929779	5.441178	-4.608663
H	-1.265015	3.097249	-5.154212
O	-3.976536	1.502590	0.843537
H	-3.265250	0.913895	0.493608
H	-3.649162	1.767074	1.717297

TS5kA-H_2O

C	-0.408494	2.532350	-0.698826
H	-1.077685	2.665581	0.153051
C	0.208641	3.920989	-0.866535
C	0.763483	4.421095	-2.054907
C	0.199341	4.764526	0.258403
C	1.283202	5.717569	-2.116979
H	0.812936	3.798989	-2.948177
C	0.728904	6.056618	0.205676

H	-0.233764	4.400257	1.194398
C	1.270708	6.541212	-0.987621
H	1.706250	6.082687	-3.056355
H	0.707239	6.688064	1.097584
H	1.678477	7.553906	-1.038536
H	0.644293	-1.213299	-1.501650
C	1.348174	-1.784743	-0.880061
H	0.826330	-2.656694	-0.460483
H	2.169112	-2.138974	-1.520289
O	0.860364	-0.500691	1.117879
O	2.595677	0.204649	-0.276612
C	1.918253	-0.931659	0.247559
C	0.064717	0.518671	0.706736
C	1.852863	1.259639	-0.780812
O	-1.050717	0.523439	1.323727
O	2.434341	1.982048	-1.579963
C	0.522180	1.413958	-0.267671
C	2.908599	-1.679815	1.114907
H	2.423282	-2.553272	1.572810
H	3.754930	-2.023039	0.503672
H	3.282745	-1.016737	1.908270
H	-3.168538	2.450877	0.437479
N	-3.348587	3.232704	-0.916357
H	-1.858046	1.146113	-1.444967
H	-4.080987	3.932645	-1.067129
C	-1.429914	2.083240	-1.830839
C	-2.573982	3.068724	-1.924163
C	-0.806130	1.702067	-3.156956
O	-0.053908	0.751494	-3.283858
N	-2.711061	3.772756	-3.100661

H	-3.431681	4.492493	-3.161925
N	-1.141195	2.475336	-4.248667
C	-2.044519	3.522976	-4.282340
O	-2.254307	4.165313	-5.294078
H	-0.701403	2.240583	-5.139242
O	-2.991225	1.938702	1.330797
H	-1.995273	1.297302	1.257934
H	-2.835063	2.628902	1.997776

MS7A-H_2O

C	-0.457840	2.564236	-0.675994
H	-1.120589	2.692196	0.184279
C	0.197459	3.942020	-0.809657
C	0.690963	4.490043	-2.003444
C	0.295624	4.716967	0.359455
C	1.255222	5.769330	-2.028319
H	0.651955	3.920890	-2.931790
C	0.869617	5.990548	0.342404
H	-0.088593	4.313372	1.300752
C	1.349655	6.524872	-0.856529
H	1.626767	6.174250	-2.973100
H	0.931359	6.569431	1.267425
H	1.791467	7.524228	-0.878326
H	0.522439	-1.288764	-1.300501
C	1.296864	-1.799765	-0.710801
H	0.837761	-2.633968	-0.161626
H	2.050616	-2.203313	-1.402124
O	1.002328	-0.347072	1.207847
O	2.565808	0.244956	-0.424584
C	1.972463	-0.848688	0.268381

C	0.164600	0.616789	0.791556
C	1.765741	1.255569	-0.912203
O	-0.886485	0.652865	1.562654
O	2.243068	1.938769	-1.802762
C	0.479932	1.438589	-0.277261
C	3.061558	-1.505391	1.087764
H	2.643864	-2.341880	1.665039
H	3.845130	-1.890500	0.420619
H	3.502854	-0.772181	1.777847
H	-3.256859	2.389185	0.388353
N	-3.316888	3.487842	-0.945700
H	-1.998361	1.275334	-1.388471
H	-3.987981	4.238749	-1.134364
C	-1.493382	2.159493	-1.804303
C	-2.555324	3.231984	-1.941293
C	-0.900711	1.675247	-3.111877
O	-0.244196	0.652113	-3.198663
N	-2.621646	3.903544	-3.148461
H	-3.278972	4.678396	-3.240043
N	-1.158651	2.436084	-4.232323
C	-1.974089	3.554133	-4.312782
O	-2.122334	4.170156	-5.352210
H	-0.743140	2.127042	-5.111845
O	-3.155799	1.656518	1.068860
H	-1.729471	1.171522	1.268023
H	-3.604352	0.898359	0.660779

TS5kB-H_2O

C	-0.685373	2.297509	-1.165558
H	0.041092	2.801533	-1.816337

C	-0.651675	3.108763	0.130763
C	-0.814450	2.548447	1.407217
C	-0.383954	4.486953	0.044634
C	-0.733784	3.343737	2.555441
H	-0.998996	1.480729	1.522214
C	-0.307051	5.284741	1.188402
H	-0.223255	4.940017	-0.937486
C	-0.485442	4.714825	2.452552
H	-0.863384	2.882626	3.537966
H	-0.095909	6.352796	1.091263
H	-0.420576	5.333237	3.351238
H	1.530025	-0.112374	0.977726
C	1.766526	-1.147723	0.693862
H	2.857027	-1.243271	0.592519
H	1.425799	-1.820501	1.493708
O	1.546968	-0.690295	-1.680495
O	-0.324816	-1.432406	-0.507001
C	1.100861	-1.536038	-0.620857
C	1.044763	0.583758	-1.769773
C	-0.881397	-0.194401	-0.507240
O	1.665831	1.361920	-2.484703
O	-2.056892	-0.231544	0.003938
C	-0.184100	0.872342	-1.066901
C	1.397592	-2.960836	-1.037153
H	2.481229	-3.093137	-1.162849
H	1.041241	-3.658778	-0.266618
H	0.893875	-3.183371	-1.988747
H	-3.742152	2.261119	0.209145
N	-3.691859	3.594875	-0.684530
H	-2.678687	1.494941	-1.707527

H	-4.183631	4.483483	-0.553254
C	-2.070914	2.363262	-1.979589
C	-2.893758	3.590292	-1.685019
C	-1.720557	2.214793	-3.443435
O	-1.450639	1.144247	-3.956839
N	-2.701800	4.674368	-2.519262
H	-3.140803	5.563893	-2.280727
N	-1.640464	3.391486	-4.161321
C	-2.087419	4.638117	-3.754471
O	-1.977477	5.626791	-4.455881
H	-1.291139	3.337173	-5.118653
O	-3.718516	1.355985	0.700670
H	-2.783086	0.623786	0.279787
H	-4.544354	0.909060	0.450201

MS7B-H_2O

C	-0.724356	2.305098	-1.115311
H	0.000579	2.811610	-1.764462
C	-0.681594	3.106258	0.187232
C	-0.868717	2.548348	1.461672
C	-0.376574	4.477233	0.107101
C	-0.773517	3.338465	2.612356
H	-1.082985	1.486471	1.577175
C	-0.284692	5.269779	1.253308
H	-0.198421	4.928091	-0.872883
C	-0.486352	4.702142	2.514838
H	-0.921539	2.878272	3.592642
H	-0.043361	6.331720	1.160049
H	-0.410243	5.316115	3.415684
H	1.592691	-0.089663	0.936437

C	1.842588	-1.116842	0.634545
H	2.928843	-1.183591	0.480065
H	1.557559	-1.803311	1.444367
O	1.509373	-0.663435	-1.727855
O	-0.296249	-1.426467	-0.471174
C	1.126542	-1.515357	-0.648795
C	0.986874	0.599387	-1.796580
C	-0.859893	-0.202667	-0.454610
O	1.554182	1.385499	-2.542421
O	-2.021195	-0.282871	0.129318
C	-0.217620	0.877776	-1.033867
C	1.417006	-2.936188	-1.079522
H	2.495505	-3.058260	-1.250733
H	1.099644	-3.637347	-0.295152
H	0.875440	-3.162024	-2.009249
H	-3.829208	2.289419	0.214772
N	-3.693612	3.731019	-0.709579
H	-2.753179	1.556934	-1.606647
H	-4.146294	4.646926	-0.631351
C	-2.107480	2.379804	-1.925341
C	-2.879850	3.653043	-1.692638
C	-1.778348	2.141849	-3.383949
O	-1.568115	1.034808	-3.846094
N	-2.618615	4.691956	-2.570201
H	-3.014151	5.611748	-2.376605
N	-1.654987	3.274733	-4.161510
C	-2.024324	4.563280	-3.807475
O	-1.867056	5.509035	-4.558362
H	-1.323426	3.156754	-5.119074
O	-3.837061	1.371613	0.626920

H	-2.670918	0.520926	0.247108
H	-4.608377	0.938821	0.226868

MS7A

C	-0.510126	2.560995	-0.674555
H	-1.222440	2.665137	0.153851
C	0.134189	3.949286	-0.754391
C	0.606029	4.552268	-1.929354
C	0.261760	4.661539	0.451109
C	1.179593	5.828030	-1.900550
H	0.545892	4.027438	-2.882746
C	0.842963	5.930775	0.486099
H	-0.103459	4.209036	1.378013
C	1.302596	6.522340	-0.694604
H	1.535933	6.277550	-2.830918
H	0.928691	6.461697	1.437625
H	1.751865	7.518375	-0.674470
H	0.326140	-1.269157	-1.166988
C	1.144482	-1.785405	-0.646532
H	0.726286	-2.584203	-0.017939
H	1.805009	-2.237139	-1.400339
O	1.112056	-0.253870	1.238189
O	2.506207	0.224698	-0.570930
C	1.956582	-0.829339	0.214231
C	0.286906	0.724081	0.869530
C	1.692742	1.241668	-1.013921
O	-0.661597	0.862273	1.782922
O	2.106282	1.905143	-1.947607
C	0.463091	1.463982	-0.277195
C	3.104012	-1.496501	0.938235

H	2.722357	-2.294737	1.589888
H	3.795726	-1.935405	0.206033
H	3.642498	-0.756499	1.547122
N	-3.269921	3.626226	-1.078390
H	-2.061832	1.305767	-1.414961
H	-3.893069	4.393264	-1.354481
C	-1.505544	2.157579	-1.834451
C	-2.520869	3.266613	-2.046914
C	-0.899389	1.585619	-3.102526
O	-0.304355	0.521321	-3.122794
N	-2.552322	3.850445	-3.310853
H	-3.182151	4.638682	-3.459603
N	-1.086807	2.301276	-4.264055
C	-1.883146	3.426283	-4.431158
O	-1.989071	3.979345	-5.512586
H	-0.668105	1.925974	-5.115596
H	-1.221785	1.637252	1.610549

MS7B

C	-0.878736	2.380056	-1.275501
H	-0.316981	2.974041	-2.009593
C	-0.615922	3.005429	0.093747
C	-1.128470	2.424955	1.265409
C	0.165838	4.163322	0.200294
C	-0.866878	2.993339	2.513607
H	-1.734981	1.517628	1.200008
C	0.433806	4.731807	1.450947
H	0.573546	4.621819	-0.704407
C	-0.083199	4.149755	2.610483
H	-1.274153	2.531052	3.416422

H	1.049629	5.632485	1.516015
H	0.124520	4.591805	3.588174
H	0.871428	0.167647	1.155446
C	1.388643	-0.780201	0.949257
H	2.450927	-0.664940	1.207425
H	0.954203	-1.565791	1.583707
O	1.849624	-0.194368	-1.365240
O	-0.117898	-1.343025	-0.871444
C	1.270023	-1.183243	-0.513427
C	1.183722	0.988092	-1.551363
C	-0.840924	-0.233874	-1.077515
O	1.836864	1.942089	-1.941551
O	-2.130218	-0.512940	-1.048822
C	-0.253101	0.986221	-1.321095
C	1.947271	-2.497398	-0.832319
H	3.018416	-2.428276	-0.597593
H	1.502694	-3.302517	-0.230889
H	1.824575	-2.731336	-1.899472
N	-3.359578	4.507094	-0.642679
H	-3.027724	1.996410	-0.950125
H	-3.541348	5.506120	-0.796874
C	-2.398590	2.524830	-1.675562
C	-2.810799	3.981463	-1.665051
C	-2.638893	1.901998	-3.032497
O	-2.889927	0.704856	-3.172347
N	-2.488018	4.678163	-2.836391
H	-2.564784	5.695423	-2.831773
N	-2.512497	2.720263	-4.112516
C	-2.377292	4.111853	-4.079317
O	-2.212202	4.753046	-5.099820

H	-2.565359	2.300565	-5.041943
H	-2.593945	-0.039809	-1.809983

MS8A

C	-0.470913	2.670127	-0.509522
H	-1.063532	2.756135	0.408782
C	0.240638	4.016481	-0.642265
C	0.563989	4.627530	-1.863060
C	0.569111	4.694804	0.543736
C	1.192589	5.876078	-1.895807
H	0.341798	4.130127	-2.808046
C	1.208826	5.936592	0.515830
H	0.312908	4.243479	1.506938
C	1.520857	6.535403	-0.707983
H	1.429723	6.332868	-2.859992
H	1.453074	6.441512	1.453911
H	2.013462	7.510516	-0.736089
H	1.347732	-2.440036	-1.675498
C	1.998764	-2.361010	-0.793266
H	1.741221	-3.153592	-0.077397
H	3.046150	-2.484022	-1.101496
O	0.418284	-0.914975	0.199856
O	2.139804	-0.033386	-1.151609
C	1.813823	-1.005329	-0.145979
C	-0.241867	0.249511	0.259682
C	1.596378	1.197226	-1.202020
O	-1.376277	0.258036	0.676788
O	2.034150	1.985622	-2.002552
C	0.500365	1.496450	-0.192600
C	2.673054	-0.796710	1.089126

H	2.430301	-1.564835	1.836669
H	3.733005	-0.890840	0.814331
H	2.511357	0.194798	1.536049
N	-3.042802	4.097714	-0.824640
H	-2.194889	1.578080	-1.101321
H	-3.641498	4.873240	-1.129648
C	-1.564270	2.340636	-1.586119
C	-2.483754	3.526571	-1.818962
C	-1.084967	1.622531	-2.830578
O	-0.475092	0.565106	-2.765281
N	-2.677874	3.912358	-3.144218
H	-3.281643	4.714513	-3.323603
N	-1.406971	2.171430	-4.045493
C	-2.185427	3.300818	-4.267978
O	-2.420024	3.699821	-5.395595
H	-1.091607	1.678024	-4.881253
H	1.058297	1.816169	0.710783

MS8B

C	-0.951179	2.297179	-1.481919
H	-0.379152	2.931229	-2.173044
C	-0.629680	2.763218	-0.067470
C	-1.360926	2.332658	1.053020
C	0.464017	3.619459	0.135959
C	-1.002659	2.744913	2.338456
H	-2.222337	1.675643	0.926717
C	0.825439	4.030477	1.422971
H	1.041992	3.965881	-0.724617
C	0.092734	3.593488	2.529288
H	-1.586505	2.402976	3.196732

H	1.680612	4.697284	1.558252
H	0.369587	3.915590	3.536217
H	1.089696	0.782538	1.111664
C	1.583863	-0.199394	1.087937
H	2.668166	-0.058732	1.199732
H	1.214627	-0.805604	1.927146
O	1.800014	-0.121952	-1.312217
O	-0.122246	-1.126990	-0.343118
C	1.296230	-0.913278	-0.220858
C	1.109405	0.872547	-1.887835
C	-0.974592	-0.236615	-0.873526
O	1.695238	1.681915	-2.566752
O	-2.161325	-0.397398	-0.710473
C	-0.404484	0.858793	-1.752871
C	1.946272	-2.273657	-0.340051
H	3.037378	-2.169891	-0.263896
H	1.592269	-2.924569	0.471261
H	1.689924	-2.726573	-1.308348
N	-3.482231	4.266049	-0.571087
H	-3.085445	1.819116	-1.248674
H	-3.688131	5.272495	-0.583932
C	-2.468099	2.473853	-1.871810
C	-2.911021	3.905526	-1.652127
C	-2.674166	2.041041	-3.314346
O	-2.878418	0.883713	-3.640914
N	-2.596464	4.774894	-2.703732
H	-2.708719	5.777091	-2.553098
N	-2.545397	3.034214	-4.258209
C	-2.462932	4.401876	-4.017811
O	-2.316590	5.201407	-4.926441

H	-2.569701	2.757836	-5.240290
H	-0.731573	0.557136	-2.765156

TS8A1

C	-0.513150	2.489467	-0.278811
H	-1.006908	2.551843	0.702116
C	0.155826	3.841449	-0.519882
C	0.291368	4.441821	-1.780163
C	0.695632	4.506648	0.593454
C	0.950444	5.666716	-1.921920
H	-0.118156	3.959731	-2.669242
C	1.359851	5.727889	0.454791
H	0.590390	4.060374	1.586712
C	1.490833	6.314212	-0.807331
H	1.041088	6.116747	-2.913887
H	1.769551	6.225199	1.337700
H	2.006058	7.271251	-0.921191
H	1.603909	-2.704272	-1.348739
C	2.314678	-2.400777	-0.566863
H	2.265061	-3.119825	0.262896
H	3.332892	-2.405942	-0.980802
O	0.630348	-1.069715	0.394379
O	2.038232	-0.151749	-1.242465
C	1.960122	-1.013971	-0.062287
C	-0.141954	0.137526	0.586878
C	1.292761	0.958536	-1.343685
O	-0.616356	0.283614	1.737089
O	1.284763	1.557070	-2.398775
C	0.527341	1.356458	-0.100487
C	2.939655	-0.525217	1.000308

H	2.916834	-1.221781	1.850757
H	3.959308	-0.506307	0.589474
H	2.695465	0.477644	1.373919
N	-1.377746	-0.210192	-0.492523
H	-1.165634	2.118497	-2.322181
H	-1.722596	-1.172707	-0.443226
C	-1.593386	2.086574	-1.299775
C	-2.054937	0.657213	-1.160950
C	-2.785955	3.037009	-1.365324
O	-2.775522	4.171602	-0.940289
N	-3.184319	0.311109	-1.842885
H	-3.453752	-0.673321	-1.894717
N	-3.886004	2.534250	-2.035349
C	-4.139521	1.205627	-2.314003
O	-5.140582	0.829129	-2.885540
H	-4.652670	3.181705	-2.225397
H	1.274598	1.717398	0.621955

MS10A1

C	-0.520300	2.490764	-0.272450
H	-1.018750	2.556970	0.706076
C	0.154274	3.839732	-0.513940
C	0.296032	4.436636	-1.775243
C	0.693464	4.505077	0.599494
C	0.960413	5.658469	-1.917823
H	-0.112783	3.954009	-2.664340
C	1.363066	5.723367	0.459985
H	0.583585	4.061498	1.593450
C	1.500147	6.306268	-0.803025
H	1.055852	6.105874	-2.910529

H	1.772222	6.221013	1.342955
H	2.019641	7.260920	-0.917572
H	1.673744	-2.677757	-1.369461
C	2.344746	-2.383151	-0.550014
H	2.264897	-3.119102	0.262443
H	3.380736	-2.370506	-0.917089
O	0.604966	-1.084312	0.350821
O	2.077092	-0.123691	-1.196717
C	1.953573	-1.009999	-0.034655
C	-0.168407	0.128077	0.579646
C	1.306529	0.967436	-1.317988
O	-0.611035	0.260039	1.748214
O	1.304220	1.562130	-2.375572
C	0.514390	1.354042	-0.088201
C	2.880933	-0.536111	1.080589
H	2.828532	-1.253470	1.912224
H	3.917111	-0.498593	0.714820
H	2.612470	0.454594	1.468795
N	-1.396362	-0.202675	-0.459036
H	-1.161359	2.104733	-2.316464
H	-1.748068	-1.162061	-0.398054
C	-1.596081	2.086334	-1.296475
C	-2.065374	0.662245	-1.141715
C	-2.784679	3.040731	-1.379647
O	-2.772312	4.179443	-0.966691
N	-3.193660	0.311020	-1.819259
H	-3.468766	-0.672718	-1.856643
N	-3.882244	2.534843	-2.051914
C	-4.141667	1.203730	-2.310887
O	-5.140807	0.822432	-2.881821

H	-4.644842	3.183432	-2.254398
H	1.246981	1.707416	0.651861

TS8A2

C	-0.308158	2.107977	-0.530363
H	-1.024240	1.955195	0.286895
C	0.113438	3.572572	-0.486737
C	1.054095	4.103837	-1.383830
C	-0.418102	4.413381	0.502678
C	1.441969	5.443709	-1.298532
H	1.497964	3.450558	-2.137777
C	-0.028370	5.753179	0.592064
H	-1.150212	4.014860	1.210192
C	0.901588	6.274594	-0.311587
H	2.175723	5.839781	-2.005423
H	-0.455492	6.390982	1.370275
H	1.207293	7.321812	-0.245188
H	1.990850	-3.068825	-0.351703
C	2.853730	-2.536561	0.074048
H	3.055610	-2.921359	1.083558
H	3.734675	-2.716940	-0.557774
O	1.368375	-0.920080	0.946778
O	2.301534	-0.631428	-1.202110
C	2.562415	-1.048508	0.125910
C	0.531896	0.120814	0.827877
C	1.461600	0.467866	-1.500710
O	-0.435475	0.174472	1.555575
O	1.830228	1.166339	-2.464641
C	0.881353	1.147313	-0.235583
C	3.701929	-0.254176	0.753831

H	3.887670	-0.610403	1.777143
H	4.613484	-0.403658	0.157673
H	3.486764	0.822417	0.789652
N	-0.008563	-0.425113	-1.954860
H	-0.467882	2.104313	-2.701159
H	0.004761	-1.435943	-2.111159
C	-1.064347	1.721874	-1.854851
C	-1.108606	0.228597	-2.067932
C	-2.413581	2.410106	-1.958761
O	-2.555581	3.606160	-1.797169
N	-2.285508	-0.371321	-2.397461
H	-2.296809	-1.378865	-2.565525
N	-3.499056	1.619718	-2.277816
C	-3.514956	0.259887	-2.508867
O	-4.525908	-0.349448	-2.790420
H	-4.400302	2.092393	-2.361218
H	1.705434	1.745869	0.183795

MS10A2

C	-0.320618	1.991636	-0.611125
H	-1.094320	1.707413	0.114255
C	0.016508	3.455968	-0.384086
C	0.997592	4.101056	-1.155194
C	-0.629811	4.177020	0.627639
C	1.316535	5.439835	-0.923482
H	1.519485	3.545551	-1.940007
C	-0.307577	5.517916	0.865471
H	-1.388711	3.682199	1.239156
C	0.663827	6.153722	0.088915
H	2.080035	5.928689	-1.533974

H	-0.818842	6.065332	1.661394
H	0.916159	7.201169	0.272224
H	1.995145	-3.006095	0.843248
C	2.929746	-2.454677	0.666203
H	3.511027	-2.420666	1.598423
H	3.517379	-2.979469	-0.100220
O	1.831034	-0.450274	1.287084
O	1.789606	-1.166156	-0.932395
C	2.613502	-1.047767	0.192902
C	0.905258	0.483057	1.032696
C	1.205621	0.050622	-1.506111
O	0.158821	0.830622	1.925093
O	1.802783	0.520036	-2.525896
C	0.917094	1.081855	-0.358779
C	3.876502	-0.224708	-0.043431
H	4.469012	-0.176272	0.881308
H	4.477705	-0.716152	-0.821802
H	3.660611	0.797523	-0.377796
N	-0.200118	-0.553426	-1.857731
H	-0.173706	1.943767	-2.789068
H	-0.299817	-1.569978	-1.896013
C	-0.938949	1.707545	-2.030583
C	-1.187921	0.232760	-2.136792
C	-2.148740	2.565816	-2.350893
O	-2.102054	3.778505	-2.387639
N	-2.404127	-0.238813	-2.483944
H	-2.555480	-1.247748	-2.548810
N	-3.331997	1.903858	-2.620424
C	-3.531139	0.544899	-2.726007
O	-4.598948	0.048298	-3.009485

H	-4.141464	2.487174	-2.839379
H	1.813931	1.717049	-0.368834

TS8B1

C	-0.950576	2.693893	-1.733167
H	-0.617669	3.618979	-2.219764
C	-0.407300	2.745181	-0.307676
C	-1.108185	2.291259	0.819062
C	0.872922	3.292536	-0.116394
C	-0.544516	2.377421	2.096945
H	-2.105941	1.857260	0.726663
C	1.441567	3.373085	1.155979
H	1.429678	3.665991	-0.980776
C	0.732594	2.914951	2.271304
H	-1.111420	2.018529	2.959568
H	2.438486	3.804213	1.277564
H	1.171453	2.981639	3.269847
H	-1.518129	-1.414435	-5.577138
C	-0.440093	-1.546858	-5.407069
H	-0.251424	-2.577784	-5.076333
H	0.100073	-1.370346	-6.347711
O	-0.756698	-0.801329	-3.200193
O	-0.267760	0.753990	-4.880077
C	0.031946	-0.575140	-4.343093
C	-0.700050	0.125935	-2.113575
C	-0.512368	1.786293	-4.067247
O	-0.272642	-0.315785	-1.040219
O	-0.827386	2.853824	-4.551717
C	-0.325689	1.552473	-2.577211
C	1.528333	-0.688201	-4.073562

H	1.748186	-1.700598	-3.705559
H	2.088546	-0.522632	-5.004899
H	1.879584	0.033701	-3.324238
N	-2.503239	0.296338	-1.937529
H	-2.696823	2.921284	-2.938740
H	-2.971092	-0.601878	-1.791459
C	-2.487458	2.702110	-1.871987
C	-3.143083	1.368146	-1.643312
C	-3.150660	3.870762	-1.166749
O	-2.611047	4.951639	-1.040010
N	-4.438889	1.363410	-1.204474
H	-4.936135	0.474113	-1.136847
N	-4.449112	3.663366	-0.752947
C	-5.142793	2.466570	-0.746785
O	-6.287873	2.379120	-0.353193
H	-4.945496	4.463395	-0.357881
H	0.765376	1.613898	-2.427190

MS10B1

C	-0.949274	2.722516	-1.738829
H	-0.624985	3.644965	-2.235557
C	-0.393390	2.774664	-0.318541
C	-1.071680	2.304720	0.815294
C	0.884771	3.332877	-0.142634
C	-0.488648	2.386575	2.085061
H	-2.068115	1.864653	0.738635
C	1.472109	3.409725	1.121158
H	1.424561	3.716963	-1.012942
C	0.785177	2.935877	2.243799
H	-1.038795	2.015962	2.953506

H	2.466532	3.849740	1.230644
H	1.238773	2.999605	3.235923
H	-1.438422	-1.251690	-5.777519
C	-0.403053	-1.467934	-5.477669
H	-0.333768	-2.517145	-5.157360
H	0.263352	-1.314397	-6.338108
O	-0.952241	-0.717953	-3.320713
O	-0.129059	0.808920	-4.873886
C	-0.001738	-0.558507	-4.330916
C	-0.858534	0.150807	-2.130084
C	-0.445207	1.824346	-4.066281
O	-0.300585	-0.380550	-1.129426
O	-0.723602	2.902739	-4.552837
C	-0.355341	1.562944	-2.571685
C	1.439548	-0.793689	-3.889200
H	1.536570	-1.829989	-3.534709
H	2.117325	-0.648118	-4.742451
H	1.751319	-0.127443	-3.075243
N	-2.424935	0.284209	-1.871147
H	-2.704241	2.898490	-2.939411
H	-2.862546	-0.624011	-1.694319
C	-2.483399	2.703873	-1.869013
C	-3.095046	1.359590	-1.616212
C	-3.183013	3.858231	-1.174476
O	-2.672444	4.952016	-1.053690
N	-4.379717	1.307235	-1.182889
H	-4.846623	0.402081	-1.097424
N	-4.477818	3.615048	-0.765969
C	-5.129406	2.397534	-0.745858
O	-6.270311	2.266996	-0.359732

H	-5.001001	4.403162	-0.380712
H	0.725980	1.538352	-2.367639

MS11A1

C	-0.558821	2.519386	-0.071046
H	-0.912216	2.638325	0.968054
C	0.125262	3.845782	-0.406212
C	0.191869	4.388784	-1.698101
C	0.745986	4.544704	0.640627
C	0.864109	5.590071	-1.934300
H	-0.282139	3.867533	-2.530478
C	1.425357	5.744781	0.407987
H	0.692632	4.144238	1.657467
C	1.486943	6.272937	-0.883677
H	0.901864	5.996398	-2.948483
H	1.900155	6.269558	1.241013
H	2.012128	7.212826	-1.071751
H	1.501021	-2.642961	-1.331860
C	2.244242	-2.371294	-0.568935
H	2.205298	-3.103273	0.249889
H	3.248271	-2.390605	-1.015151
O	0.622517	-1.044159	0.514158
O	1.969443	-0.106761	-1.177822
C	1.940970	-0.986266	-0.029556
C	-0.241053	0.054538	0.456269
C	1.261998	1.041005	-1.227827
O	-0.778174	0.147498	1.746814
O	1.300144	1.691692	-2.244073
C	0.465882	1.357657	0.025152
C	2.958901	-0.535424	1.010567

H	2.932947	-1.230850	1.861532
H	3.966692	-0.553423	0.571856
H	2.759217	0.477223	1.384497
N	-1.250330	-0.236724	-0.548248
H	-1.590874	-1.196617	-0.571750
C	-1.750410	2.101731	-0.916062
C	-2.021348	0.753000	-1.082583
C	-2.752967	3.042893	-1.346547
O	-2.773443	4.251888	-1.101359
N	-3.109744	0.329763	-1.808831
H	-3.284351	-0.666578	-1.930866
N	-3.800931	2.503797	-2.112674
C	-4.042253	1.180034	-2.364515
O	-5.000194	0.772031	-3.008392
H	-4.504772	3.162416	-2.442697
H	1.185041	1.610570	0.815975
H	-1.672107	0.521384	1.712112

MS11A2

C	-0.446504	2.056100	-0.152637
H	-0.973273	2.126908	0.809507
C	0.045566	3.471927	-0.472425
C	0.013222	4.009103	-1.766237
C	0.542235	4.270235	0.571165
C	0.473549	5.306993	-2.013601
H	-0.376359	3.409364	-2.592004
C	1.003937	5.566004	0.327991
H	0.562338	3.871705	1.589996
C	0.971189	6.090129	-0.968747
H	0.440325	5.707301	-3.030266

H	1.383153	6.171308	1.155387
H	1.327658	7.105050	-1.161935
H	1.854056	-3.162953	-0.190806
C	2.717835	-2.616911	0.213924
H	2.963581	-3.011957	1.209304
H	3.581371	-2.762172	-0.449995
O	1.220693	-1.055735	1.171808
O	2.021997	-0.730210	-0.994921
C	2.391054	-1.139115	0.308413
C	0.350695	-0.038619	1.074095
C	1.322675	0.481548	-1.203094
O	-0.625524	-0.028020	1.788076
O	2.187735	1.448220	-1.715294
C	0.715997	1.062987	0.091190
C	3.544182	-0.332955	0.894952
H	3.792706	-0.723226	1.892021
H	4.422778	-0.446583	0.244169
H	3.321450	0.737288	0.986223
N	0.287813	0.116334	-2.153154
H	2.517807	1.142794	-2.577307
H	0.525975	-0.636065	-2.796323
C	-1.410637	1.492519	-1.159643
C	-0.983952	0.576513	-2.098634
C	-2.773027	1.935377	-1.181257
O	-3.272048	2.758861	-0.406295
N	-1.858239	0.077664	-3.037053
H	-1.541510	-0.604693	-3.723970
N	-3.575793	1.364720	-2.181237
C	-3.185643	0.446598	-3.121833
O	-3.938405	-0.014209	-3.970350

H	-4.550097	1.660826	-2.216471
H	1.532375	1.611789	0.582179

MS11B1

C	-0.998013	2.678055	-1.537199
H	-0.757886	3.608043	-2.071978
C	-0.272307	2.761231	-0.190487
C	-0.844651	2.307970	1.005576
C	1.018339	3.314039	-0.147344
C	-0.141986	2.394680	2.212496
H	-1.851029	1.883076	0.998085
C	1.723745	3.399983	1.055066
H	1.475059	3.684868	-1.069860
C	1.144811	2.938709	2.242326
H	-0.606019	2.035105	3.134658
H	2.726419	3.835204	1.066428
H	1.692419	3.007961	3.185583
H	-1.523269	-1.239706	-5.653723
C	-0.470002	-1.422246	-5.397900
H	-0.348664	-2.469898	-5.089260
H	0.156077	-1.237102	-6.281620
O	-0.946894	-0.754234	-3.199475
O	-0.285463	0.847714	-4.757708
C	-0.051413	-0.504653	-4.265562
C	-1.043944	0.151116	-2.116257
C	-0.553694	1.858891	-3.917343
O	-0.346003	-0.343570	-1.013899
O	-0.800451	2.952762	-4.370905
C	-0.452375	1.540619	-2.434058
C	1.413928	-0.690032	-3.888852

H	1.568569	-1.731672	-3.573292
H	2.044527	-0.492515	-4.767226
H	1.738065	-0.033367	-3.072128
N	-2.472186	0.225807	-1.869065
H	-3.002423	-0.608453	-2.112164
C	-2.495662	2.588215	-1.436146
C	-3.133880	1.377236	-1.602004
C	-3.272623	3.754246	-1.134879
O	-2.820753	4.890206	-0.953341
N	-4.503584	1.287884	-1.503323
H	-4.968289	0.389504	-1.625505
N	-4.657891	3.547436	-1.045401
C	-5.323099	2.360145	-1.214417
O	-6.538299	2.245928	-1.120832
H	-5.233399	4.359700	-0.828473
H	0.627584	1.451827	-2.247898
H	-0.747368	-1.188241	-0.747144

TS10A1

C	-0.621579	2.629369	-0.015948
H	-0.970852	2.773674	1.021587
C	0.088140	3.921994	-0.407939
C	0.374974	4.255580	-1.741873
C	0.527242	4.786761	0.605677
C	1.077107	5.423406	-2.048866
H	0.046001	3.595637	-2.545855
C	1.233037	5.955135	0.301151
H	0.308940	4.543690	1.649504
C	1.510570	6.278259	-1.029450
H	1.285679	5.668714	-3.093569

H	1.562579	6.615155	1.107708
H	2.058902	7.191924	-1.272500
H	1.883781	-2.967992	-1.401850
C	2.589575	-2.424559	-0.761264
H	3.217739	-3.107043	-0.171901
H	3.262376	-1.823757	-1.401863
O	0.741484	-1.099813	-0.255235
O	2.503030	1.314128	-1.124023
C	1.886576	-1.445201	0.110467
C	-0.284944	0.187648	0.427493
C	1.258517	1.278868	-1.272360
O	-0.291711	-0.077338	1.730463
O	0.630582	1.081270	-2.336535
C	0.423300	1.467258	0.043636
C	2.587830	-0.957575	1.325213
H	2.573039	-1.795144	2.046884
H	3.645104	-0.779945	1.079886
H	2.136585	-0.079989	1.793186
N	-1.433227	-0.091591	-0.297099
H	-1.846501	-1.019970	-0.203673
C	-1.820591	2.227424	-0.855130
C	-2.139424	0.894385	-0.951975
C	-2.755031	3.181450	-1.401297
O	-2.708899	4.405682	-1.268272
N	-3.226762	0.454402	-1.661126
H	-3.425967	-0.542428	-1.736110
N	-3.817048	2.628710	-2.135987
C	-4.113382	1.301121	-2.296353
O	-5.073441	0.887442	-2.931478
H	-4.474473	3.290129	-2.546961

H	1.124437	1.700660	0.851436
H	-0.950546	-0.758041	1.962401

MS12A1

C	-0.892860	2.847584	0.038446
H	-1.237452	3.048354	1.067678
C	-0.097019	4.075535	-0.395294
C	0.214299	4.327161	-1.741163
C	0.377911	4.961908	0.581115
C	0.976332	5.441415	-2.098334
H	-0.146129	3.647879	-2.516645
C	1.144960	6.076384	0.226163
H	0.139194	4.780576	1.632885
C	1.445511	6.320781	-1.116184
H	1.203915	5.625152	-3.151550
H	1.503531	6.757256	1.002348
H	2.040380	7.193301	-1.397687
H	1.921786	-4.490344	-1.383923
C	2.703001	-3.949169	-0.833456
H	2.847973	-4.391733	0.165204
H	3.664261	-4.049844	-1.366188
O	1.418178	-2.004622	-1.334618
O	2.146982	1.668134	-1.044321
C	2.358288	-2.488108	-0.719514
C	-0.713134	0.341321	0.275572
C	0.944405	1.392840	-1.195527
O	-0.147517	-0.625515	0.917727
O	0.340263	1.000301	-2.211895
C	0.035065	1.597316	0.123376
C	3.213897	-1.654100	0.198210

H	2.880693	-1.833752	1.235703
H	4.271397	-1.954244	0.144727
H	3.100704	-0.585949	-0.030282
N	-1.888038	0.186111	-0.313771
H	-2.344903	-0.731392	-0.301631
C	-2.124614	2.541386	-0.796098
C	-2.549684	1.255131	-0.938619
C	-3.003035	3.586258	-1.302137
O	-2.856909	4.790532	-1.121045
N	-3.669670	0.903332	-1.639081
H	-3.934703	-0.075822	-1.746457
N	-4.102974	3.132700	-2.038244
C	-4.499492	1.832848	-2.238359
O	-5.491367	1.514010	-2.873425
H	-4.719753	3.851628	-2.416395
H	0.720993	1.697412	0.970716
H	-0.635437	-1.473774	0.894972

MS13A1

C	-0.752595	2.768358	0.125510
H	-1.177014	3.052577	1.103701
C	0.025950	3.982004	-0.376982
C	0.381141	4.128652	-1.727341
C	0.434735	4.962952	0.536141
C	1.121948	5.233329	-2.152040
H	0.074349	3.372491	-2.453496
C	1.180364	6.068688	0.113939
H	0.160468	4.863679	1.590193
C	1.525212	6.208146	-1.232868
H	1.384979	5.334071	-3.208226

H	1.487383	6.825290	0.840474
H	2.103260	7.073420	-1.566903
O	2.416225	1.793132	-0.560025
C	-0.479770	0.276438	0.520701
C	1.282224	1.348690	-0.793450
O	0.091861	-0.644372	1.224557
O	0.844392	0.772679	-1.810886
C	0.210479	1.568775	0.392214
N	-1.596036	0.034452	-0.146617
H	-1.990306	-0.912004	-0.146575
C	-1.911016	2.348469	-0.761291
C	-2.265771	1.037719	-0.861831
C	-2.808488	3.322664	-1.368129
O	-2.733152	4.539430	-1.234736
N	-3.324092	0.597757	-1.606660
H	-3.538186	-0.397005	-1.678257
N	-3.835115	2.779962	-2.147561
C	-4.152961	1.453240	-2.308134
O	-5.081346	1.053174	-2.991093
H	-4.455614	3.446689	-2.606716
H	0.785261	1.748056	1.307000
H	-0.360851	-1.512221	1.204446

TS10A2

C	-0.529137	2.126608	-0.103983
H	-1.072503	2.214590	0.848045
C	-0.015930	3.525810	-0.448459
C	0.086411	3.990603	-1.767727
C	0.393954	4.375063	0.593104

C	0.590350	5.267458	-2.039323
H	-0.235061	3.354037	-2.595234
C	0.898296	5.649530	0.325369
H	0.312201	4.031371	1.628504
C	0.998749	6.101151	-0.995151
H	0.661285	5.611369	-3.074490
H	1.208182	6.295234	1.151085
H	1.389865	7.099323	-1.207574
H	2.278129	-3.430377	-0.300425
C	2.882541	-2.793951	0.357949
H	2.491894	-2.872958	1.389102
H	3.935097	-3.109892	0.377221
O	1.065015	-0.372588	1.997099
O	1.780401	-1.050840	-0.729624
C	2.757187	-1.357401	-0.011727
C	0.193763	-0.025882	1.162343
C	1.187944	0.591660	-1.133091
O	-0.959386	-0.488955	1.042169
O	2.280231	1.218498	-1.557886
C	0.631148	1.120925	0.176266
C	3.811535	-0.423261	0.462743
H	4.123065	-0.713262	1.475852
H	4.684471	-0.591886	-0.195441
H	3.539914	0.633402	0.418486
N	0.254459	0.211954	-2.084176
H	2.485736	1.012014	-2.488741
H	0.600028	-0.238878	-2.932533
C	-1.485515	1.558536	-1.118457
C	-1.053141	0.644940	-2.039044
C	-2.859320	1.985312	-1.157762

O	-3.372052	2.806627	-0.394717
N	-1.891449	0.127364	-2.993033
H	-1.555047	-0.563306	-3.662692
N	-3.635135	1.400923	-2.167709
C	-3.221075	0.486775	-3.105068
O	-3.953984	0.021300	-3.966119
H	-4.612098	1.687127	-2.221445
H	1.453970	1.656674	0.661205

MS12A2

C	-0.423127	2.259857	-0.263982
H	-0.756978	2.192755	0.781712
C	-0.059007	3.721053	-0.527788
C	-0.290468	4.350363	-1.758657
C	0.557722	4.455189	0.499116
C	0.089106	5.682333	-1.960470
H	-0.776817	3.803455	-2.569895
C	0.938488	5.783337	0.299069
H	0.738703	3.977267	1.466364
C	0.705317	6.402369	-0.934185
H	-0.101072	6.157376	-2.926294
H	1.413508	6.339877	1.111006
H	0.999547	7.443017	-1.091701
H	3.301878	-4.105825	0.185617
C	3.197591	-3.251180	0.867973
H	2.129682	-3.104469	1.102198
H	3.717329	-3.446945	1.818677
O	0.272120	0.215168	1.727272
O	3.930224	-1.935239	-0.983016
C	3.702854	-1.989965	0.216900

C	0.557562	-0.002494	0.539309
C	1.068192	0.839191	-1.725051
O	0.707312	-1.096133	-0.050207
O	2.290729	0.583247	-2.044615
C	0.786411	1.300792	-0.349678
C	3.920919	-0.800140	1.115675
H	3.084129	-0.667459	1.818500
H	4.823427	-0.984591	1.724636
H	4.071813	0.110014	0.519765
N	0.072839	0.665437	-2.582673
H	2.415682	0.201450	-2.938388
H	0.265342	0.268074	-3.508383
C	-1.529153	1.730722	-1.139137
C	-1.248711	0.983320	-2.240709
C	-2.919670	2.055452	-0.871125
O	-3.308880	2.733662	0.074218
N	-2.211998	0.509530	-3.086542
H	-1.966277	-0.080428	-3.881948
N	-3.832718	1.535706	-1.790884
C	-3.558448	0.767203	-2.900408
O	-4.415611	0.343569	-3.657298
H	-4.817676	1.744976	-1.627794
H	1.694961	1.777464	0.042551

MS13A2

C	-0.518905	2.227170	-0.137345
H	-1.035361	2.256423	0.833143
C	-0.086201	3.656682	-0.463377
C	-0.099047	4.175324	-1.765679
C	0.363090	4.478922	0.583614

C	0.330172	5.483622	-2.016597
H	-0.451012	3.559542	-2.596809
C	0.794596	5.783230	0.334925
H	0.372046	4.089369	1.605776
C	0.778715	6.291409	-0.968896
H	0.310689	5.871647	-3.038220
H	1.138445	6.408173	1.163095
H	1.111531	7.313677	-1.165226
O	0.652887	-1.084596	0.689883
C	0.256346	0.043564	1.049242
C	1.174848	0.668905	-1.164542
O	-0.393283	0.366233	2.056582
O	2.433727	0.400769	-1.247226
C	0.661751	1.258035	0.087999
N	0.340935	0.401272	-2.161397
H	2.707638	-0.042183	-2.076023
H	0.691326	-0.070481	-3.001790
C	-1.461738	1.626117	-1.146087
C	-1.009629	0.771764	-2.103462
C	-2.864108	2.003810	-1.173525
O	-3.394154	2.777424	-0.382350
N	-1.816611	0.244809	-3.073849
H	-1.452769	-0.412901	-3.763453
N	-3.610205	1.419715	-2.198411
C	-3.163455	0.548724	-3.167207
O	-3.881392	0.078261	-4.033589
H	-4.600281	1.662256	-2.241634
H	1.498209	1.764289	0.589967

TS10B1

C	-0.970495	2.776137	-1.510478
H	-0.734259	3.712607	-2.036029
C	-0.257005	2.841416	-0.160302
C	-0.719602	2.152561	0.971443
C	0.921489	3.597264	-0.047291
C	-0.020215	2.213367	2.181119
H	-1.638770	1.565065	0.914050
C	1.621800	3.660782	1.159887
H	1.290418	4.147832	-0.917558
C	1.152849	2.966889	2.280215
H	-0.397525	1.670313	3.051524
H	2.533710	4.259686	1.227731
H	1.696549	3.017628	3.226895
H	-1.464957	-1.720091	-5.762356
C	-0.391661	-1.552262	-5.608787
H	0.199601	-2.449030	-5.842162
H	-0.054041	-0.745353	-6.285157
O	-1.082393	-0.595780	-3.599173
O	0.368816	1.667022	-4.693058
C	-0.109584	-1.062755	-4.232570
C	-1.050778	0.308948	-2.065160
C	-0.593511	1.992914	-3.956118
O	-0.307744	-0.452239	-1.265573
O	-1.652453	2.555879	-4.303737
C	-0.419202	1.641633	-2.429992
C	1.276285	-1.198497	-3.713571
H	1.382020	-2.255128	-3.403209
H	1.988951	-1.038364	-4.534370
H	1.506251	-0.559629	-2.858352
N	-2.418837	0.326604	-1.840670

H	-2.894998	-0.568901	-1.729186
C	-2.470284	2.688530	-1.408042
C	-3.107472	1.492222	-1.588158
C	-3.259649	3.857728	-1.123412
O	-2.812809	4.992558	-0.942444
N	-4.471668	1.379311	-1.502777
H	-4.926956	0.477789	-1.639522
N	-4.641830	3.638940	-1.048363
C	-5.301388	2.446553	-1.217711
O	-6.515084	2.325670	-1.130693
H	-5.226060	4.447985	-0.840522
H	0.655253	1.530475	-2.250097
H	-0.816660	-1.191776	-0.882702

MS12B1

C	-0.516003	3.288681	-1.069980
H	-0.188462	4.307504	-1.321565
C	-0.327653	3.110099	0.435702
C	-1.160759	2.292358	1.212303
C	0.743368	3.766921	1.064426
C	-0.926824	2.131145	2.582531
H	-2.006204	1.776944	0.750125
C	0.979113	3.607343	2.431498
H	1.397789	4.413440	0.472472
C	0.143272	2.786678	3.196842
H	-1.587868	1.490213	3.171609
H	1.815148	4.130484	2.902825
H	0.323863	2.662693	4.267547
H	-1.105062	-4.426850	-3.704777
C	-0.908316	-3.794266	-4.579678

H	-0.066345	-4.215768	-5.156615
H	-1.775746	-3.774192	-5.257521
O	-0.276699	-2.173964	-2.978694
O	0.666901	2.216666	-4.321173
C	-0.536143	-2.409038	-4.166412
C	-0.169593	1.020084	-2.140671
C	0.690808	3.006878	-3.352404
O	0.609217	0.021314	-2.244421
O	0.936904	4.225260	-3.351771
C	0.393611	2.373710	-1.917061
C	-0.502989	-1.345252	-5.215630
H	-0.078879	-1.737642	-6.151799
H	-1.549727	-1.067008	-5.434204
H	0.038955	-0.450247	-4.883636
N	-1.495464	0.889135	-2.277467
H	-1.883117	-0.022581	-2.536375
C	-1.938857	3.134380	-1.539464
C	-2.363649	1.969881	-2.104195
C	-2.903878	4.202137	-1.360963
O	-2.657932	5.292073	-0.851256
N	-3.653474	1.777290	-2.520202
H	-3.939487	0.897291	-2.949330
N	-4.192475	3.915316	-1.818550
C	-4.633640	2.744311	-2.391268
O	-5.783312	2.563923	-2.759005
H	-4.893938	4.646726	-1.702849
H	1.377145	2.250554	-1.441899
H	0.182503	-0.907504	-2.557677

MS13B1

C	-0.952379	2.803584	-1.412285
H	-0.709829	3.700736	-1.999704
C	-0.270423	2.949452	-0.053431
C	-0.771460	2.347666	1.110098
C	0.927476	3.678135	0.028111
C	-0.087179	2.464765	2.324554
H	-1.706280	1.782921	1.075402
C	1.612151	3.796362	1.239708
H	1.325287	4.158258	-0.870529
C	1.106802	3.187668	2.393468
H	-0.491788	1.988127	3.221105
H	2.541441	4.370072	1.284441
H	1.639949	3.280243	3.342949
O	-0.936066	0.911286	-4.503407
C	-1.048448	0.343721	-1.959090
C	-0.573008	1.897540	-3.829449
O	-0.348197	-0.735750	-2.050385
O	-0.305030	3.050500	-4.201671
C	-0.399019	1.634844	-2.257114
N	-2.335116	0.310606	-1.634898
H	-2.804704	-0.593003	-1.517264
C	-2.453053	2.693477	-1.339800
C	-3.073449	1.487671	-1.447616
C	-3.276051	3.866509	-1.099376
O	-2.844533	5.007483	-0.967411
N	-4.430289	1.336208	-1.363102
H	-4.864801	0.418273	-1.458502
N	-4.647025	3.615771	-1.018211
C	-5.284238	2.400389	-1.134300
O	-6.492791	2.261916	-1.047046

H	-5.252267	4.419039	-0.846805
H	0.682190	1.514680	-2.100401
H	-0.844132	-1.564132	-1.886818

Propanone

H	2.405569	-4.420109	-0.110569
C	3.024287	-3.799300	0.551478
H	2.601472	-3.781841	1.568561
H	4.030806	-4.244443	0.633600
O	2.750627	-2.117472	-1.118612
C	3.150190	-2.402536	0.000291
C	3.792858	-1.374614	0.895151
H	3.124748	-1.174357	1.750233
H	4.737236	-1.757750	1.313569
H	3.969178	-0.440978	0.344108

TS11A1

C	-0.885191	2.849931	0.211452
H	-1.354067	3.256869	1.127161
C	-0.041658	3.998206	-0.353293
C	0.233842	4.131440	-1.722480
C	0.518884	4.926799	0.535992
C	1.049993	5.166027	-2.188961
H	-0.193323	3.421325	-2.433626
C	1.338707	5.960362	0.073405
H	0.305026	4.841605	1.605482
C	1.607139	6.083886	-1.293074
H	1.248712	5.256791	-3.260089
H	1.763973	6.674852	0.783014
H	2.243709	6.893527	-1.658701

O	2.473660	1.714044	-0.294993
C	-0.495405	0.373742	0.584721
C	1.444707	1.383631	-0.814785
O	0.100621	-0.597547	1.253589
O	0.928768	0.974183	-1.818851
C	0.001480	1.672424	0.638634
N	-1.525730	0.053122	-0.240344
H	-1.768340	-0.926431	-0.394557
C	-1.999510	2.372646	-0.702031
C	-2.248447	1.039161	-0.888091
C	-2.931133	3.315944	-1.284225
O	-2.921075	4.532048	-1.098372
N	-3.260279	0.587577	-1.693811
H	-3.413518	-0.411684	-1.824829
N	-3.910543	2.759359	-2.116141
C	-4.129707	1.427299	-2.362284
O	-5.013534	1.006848	-3.093631
H	-4.558059	3.410613	-2.558753
H	0.673785	1.864021	1.475504
H	-0.235567	-1.483198	1.026013

MS14A1

C	-1.078774	2.918698	0.418422
H	-1.505371	3.413524	1.308728
C	-0.166039	3.961127	-0.244216
C	0.556558	3.645408	-1.406208
C	-0.032207	5.245966	0.298804
C	1.388234	4.591339	-2.011209
H	0.466906	2.646920	-1.843606
C	0.801697	6.195677	-0.302092

H	-0.591869	5.508336	1.201135
C	1.514312	5.871857	-1.459951
H	1.941345	4.328478	-2.916878
H	0.892520	7.193307	0.135685
H	2.165374	6.612493	-1.931335
O	3.139839	1.421100	0.111662
C	-0.468681	0.487385	0.303391
C	2.646622	0.844159	-0.768156
O	0.239640	-0.607930	0.690317
O	2.166154	0.269868	-1.658020
C	-0.287271	1.700840	0.846061
N	-1.442917	0.244210	-0.675286
H	-1.644864	-0.720632	-0.929098
C	-2.224422	2.505032	-0.491909
C	-2.328493	1.227296	-0.999225
C	-3.239736	3.462804	-0.837299
O	-3.294808	4.625275	-0.423928
N	-3.350096	0.878625	-1.851480
H	-3.402318	-0.066844	-2.227912
N	-4.236076	3.004187	-1.712312
C	-4.343551	1.747468	-2.251251
O	-5.237707	1.412367	-3.017220
H	-4.961367	3.670827	-1.972944
H	0.504745	1.818572	1.586505
H	0.455611	-1.161868	-0.079246

MS15A1

C	-1.065625	2.918908	0.414383
H	-1.493617	3.410759	1.305907
C	-0.162070	3.968631	-0.248732

C	0.544553	3.665676	-1.423270
C	-0.021221	5.247638	0.306490
C	1.368693	4.617633	-2.028494
H	0.447001	2.671342	-1.868574
C	0.805072	6.203882	-0.294380
H	-0.569825	5.499406	1.218709
C	1.502531	5.892034	-1.464767
H	1.910439	4.364704	-2.943834
H	0.901803	7.196576	0.153312
H	2.148016	6.637369	-1.936438
C	-0.451793	0.486419	0.301228
O	0.255328	-0.602521	0.698494
C	-0.272302	1.702319	0.842410
N	-1.422703	0.244986	-0.681452
H	-1.626312	-0.718417	-0.940721
C	-2.212244	2.502935	-0.493283
C	-2.311997	1.226322	-1.002775
C	-3.230470	3.458176	-0.837275
O	-3.288972	4.619905	-0.422343
N	-3.332512	0.875406	-1.855460
H	-3.383079	-0.069978	-2.232425
N	-4.225493	2.997872	-1.713069
C	-4.328198	1.741995	-2.254860
O	-5.220073	1.405375	-3.022767
H	-4.951614	3.663592	-1.973755
H	0.511817	1.819595	1.591688
H	0.265226	-1.287766	0.010485

MS16A1

C	-0.750574	2.770636	0.149601

H	-1.159395	3.030187	1.141876
C	0.025835	3.985607	-0.348867
C	0.535349	4.031536	-1.656638
C	0.290320	5.059689	0.511018
C	1.284970	5.126081	-2.093298
H	0.337447	3.204470	-2.344186
C	1.042667	6.157077	0.077694
H	-0.101639	5.039170	1.531788
C	1.542082	6.194709	-1.226770
H	1.669684	5.146075	-3.116345
H	1.236081	6.986398	0.763055
H	2.128269	7.051542	-1.568426
C	-0.383613	0.225944	0.480302
O	0.129525	-0.690793	1.101116
C	0.232485	1.592250	0.330038
N	-1.554215	0.026362	-0.222640
H	-1.929067	-0.922657	-0.232967
C	-1.911014	2.359504	-0.740063
C	-2.250346	1.033560	-0.856663
C	-2.811726	3.336679	-1.310702
O	-2.726729	4.558360	-1.181100
N	-3.335999	0.622211	-1.588516
H	-3.548679	-0.370471	-1.679137
N	-3.870243	2.811473	-2.065627
C	-4.190946	1.489379	-2.237442
O	-5.145809	1.100655	-2.895128
H	-4.501204	3.487195	-2.494754
H	0.901141	1.761350	1.183810
H	0.872033	1.509614	-0.567669

TS11A2

C	-0.372352	2.215183	-0.192011
H	-0.822538	2.174808	0.811313
C	-0.038912	3.683913	-0.467012
C	0.431268	4.101069	-1.722742
C	-0.195892	4.643516	0.542976
C	0.733218	5.443850	-1.962456
H	0.558988	3.368302	-2.524706
C	0.107159	5.988788	0.307480
H	-0.566051	4.333137	1.524149
C	0.571238	6.393516	-0.947095
H	1.095374	5.751557	-2.946916
H	-0.024095	6.723099	1.106588
H	0.804369	7.444646	-1.135151
O	1.457632	-0.988061	1.111873
C	0.547472	-0.215956	1.203883
C	1.177435	0.570080	-1.297582
O	-0.456222	0.085044	1.783214
O	2.384304	0.052214	-1.423558
C	0.861728	1.326178	-0.173529
N	0.242012	0.273261	-2.246816
H	2.458253	-0.602081	-2.141801
H	0.474436	-0.376800	-2.998648
C	-1.389074	1.667585	-1.171799
C	-1.041908	0.770604	-2.144410
C	-2.757321	2.128191	-1.121716
O	-3.203649	2.926169	-0.297402
N	-1.944875	0.310639	-3.068736
H	-1.666114	-0.369041	-3.775637
N	-3.602501	1.600435	-2.104151

C	-3.268484	0.705104	-3.091562
O	-4.062843	0.285119	-3.919817
H	-4.573961	1.909873	-2.089282
H	1.728544	1.693621	0.382248

MS14A2

C	-1.078774	2.918698	0.418422
H	-1.505371	3.413524	1.308728
C	-0.166039	3.961127	-0.244216
C	0.556558	3.645408	-1.406208
C	-0.032207	5.245966	0.298804
C	1.388234	4.591339	-2.011209
H	0.466906	2.646920	-1.843606
C	0.801697	6.195677	-0.302092
H	-0.591869	5.508336	1.201135
C	1.514312	5.871857	-1.459951
H	1.941345	4.328478	-2.916878
H	0.892520	7.193307	0.135685
H	2.165374	6.612493	-1.931335
O	3.139839	1.421100	0.111662
C	-0.468681	0.487385	0.303391
C	2.646622	0.844159	-0.768156
O	0.239640	-0.607930	0.690317
O	2.166154	0.269868	-1.658020
C	-0.287271	1.700840	0.846061
N	-1.442917	0.244210	-0.675286
H	-1.644864	-0.720632	-0.929098
C	-2.224422	2.505032	-0.491909
C	-2.328493	1.227296	-0.999225
C	-3.239736	3.462804	-0.837299

O	-3.294808	4.625275	-0.423928
N	-3.350096	0.878625	-1.851480
H	-3.402318	-0.066844	-2.227912
N	-4.236076	3.004187	-1.712312
C	-4.343551	1.747468	-2.251251
O	-5.237707	1.412367	-3.017220
H	-4.961367	3.670827	-1.972944
H	0.504745	1.818572	1.586505
H	0.455611	-1.161868	-0.079246

MS15A2

C	-1.065625	2.918908	0.414383
H	-1.493617	3.410759	1.305907
C	-0.162070	3.968631	-0.248732
C	0.544553	3.665676	-1.423270
C	-0.021221	5.247638	0.306490
C	1.368693	4.617633	-2.028494
H	0.447001	2.671342	-1.868574
C	0.805072	6.203882	-0.294380
H	-0.569825	5.499406	1.218709
C	1.502531	5.892034	-1.464767
H	1.910439	4.364704	-2.943834
H	0.901803	7.196576	0.153312
H	2.148016	6.637369	-1.936438
C	-0.451793	0.486419	0.301228
O	0.255328	-0.602521	0.698494
C	-0.272302	1.702319	0.842410
N	-1.422703	0.244986	-0.681452
H	-1.626312	-0.718417	-0.940721
C	-2.212244	2.502935	-0.493283

C	-2.311997	1.226322	-1.002775
C	-3.230470	3.458176	-0.837275
O	-3.288972	4.619905	-0.422343
N	-3.332512	0.875406	-1.855460
H	-3.383079	-0.069978	-2.232425
N	-4.225493	2.997872	-1.713069
C	-4.328198	1.741995	-2.254860
O	-5.220073	1.405375	-3.022767
H	-4.951614	3.663592	-1.973755
H	0.511817	1.819595	1.591688
H	0.265226	-1.287766	0.010485

MS16A2

C	-0.556081	2.231107	-0.103759
H	-1.095896	2.298092	0.852166
C	-0.103750	3.653196	-0.448949
C	-0.101609	4.151951	-1.759418
C	0.351866	4.489291	0.584593
C	0.347539	5.449602	-2.030935
H	-0.461348	3.528325	-2.581073
C	0.800617	5.784284	0.317019
H	0.349741	4.119319	1.614159
C	0.800164	6.270391	-0.995194
H	0.338863	5.819652	-3.059465
H	1.146430	6.419150	1.136903
H	1.146968	7.284936	-1.207018
C	1.234051	0.711031	-1.143520
O	2.416530	0.445294	-1.285952
C	0.635418	1.273857	0.122166
N	0.327403	0.432253	-2.150024

H	0.688107	-0.045814	-2.976175
C	-1.489588	1.624080	-1.120439
C	-1.002047	0.792219	-2.094503
C	-2.884298	1.986207	-1.152640
O	-3.440020	2.746438	-0.358062
N	-1.811431	0.282758	-3.077766
H	-1.434838	-0.350393	-3.782199
N	-3.625315	1.409259	-2.191726
C	-3.159967	0.567883	-3.172882
O	-3.865711	0.105041	-4.057390
H	-4.616582	1.643331	-2.235573
H	1.441078	1.744174	0.699849
H	0.284977	0.396564	0.695533

TS11B1

C	-0.883295	2.683745	-1.430835
H	-0.601720	3.529620	-2.076107
C	-0.220999	2.925419	-0.071883
C	-0.461707	2.071059	1.016048
C	0.652446	4.007368	0.105146
C	0.157857	2.291551	2.248531
H	-1.143021	1.223638	0.897451
C	1.275806	4.231110	1.337455
H	0.843838	4.684568	-0.732003
C	1.029941	3.373812	2.413381
H	-0.040887	1.616160	3.084773
H	1.953478	5.080485	1.457108
H	1.514432	3.547439	3.377590
O	-0.144757	0.670870	-4.704908
C	-1.159959	0.257474	-2.036664

C	-0.367786	1.719910	-4.173581
O	-0.624721	-0.914803	-2.319222
O	-0.541343	2.896362	-4.309569
C	-0.387007	1.411815	-2.102568
N	-2.497783	0.306438	-1.771289
H	-3.053989	-0.548070	-1.816424
C	-2.395195	2.664906	-1.337843
C	-3.111420	1.508592	-1.481856
C	-3.116153	3.883780	-1.050276
O	-2.599867	4.991753	-0.904108
N	-4.476530	1.474366	-1.356214
H	-4.984792	0.597144	-1.463077
N	-4.504311	3.745833	-0.936912
C	-5.236838	2.590892	-1.069338
O	-6.452169	2.542930	-0.950339
H	-5.035005	4.591438	-0.730141
H	0.691544	1.233385	-2.086950
H	-1.278918	-1.634169	-2.384577

MS14B1

C	-0.723145	2.602149	-1.301279
H	-0.369138	3.407412	-1.967648
C	-0.148202	2.914539	0.086411
C	-0.396901	2.062342	1.173955
C	0.638022	4.055600	0.295623
C	0.124984	2.344846	2.438623
H	-1.007283	1.166856	1.026878
C	1.165624	4.341238	1.559785
H	0.835599	4.730844	-0.541786
C	0.909453	3.487301	2.636025

H	-0.080105	1.670710	3.274483
H	1.777316	5.235826	1.703783
H	1.319234	3.708982	3.624760
O	-1.361353	0.619754	-5.147558
C	-1.018995	0.205609	-1.982853
C	-1.095419	1.731229	-4.932027
O	-0.555540	-1.014940	-2.355150
O	-0.835441	2.848740	-4.743212
C	-0.222489	1.277686	-1.838913
N	-2.396147	0.268463	-1.733847
H	-2.956049	-0.578311	-1.803321
C	-2.243828	2.620934	-1.312086
C	-2.983203	1.469065	-1.480493
C	-2.943879	3.859315	-1.107617
O	-2.413635	4.966621	-0.972429
N	-4.356684	1.484716	-1.413439
H	-4.884932	0.622318	-1.537976
N	-4.343346	3.764259	-1.057953
C	-5.097782	2.626738	-1.193391
O	-6.320546	2.613481	-1.133137
H	-4.857448	4.630810	-0.906238
H	0.833318	1.170411	-2.091187
H	-1.188440	-1.452335	-2.950066

MS15B1

C	-0.834246	2.600250	-1.346496
H	-0.504053	3.407451	-2.022939
C	-0.198549	2.908414	0.016364
C	-0.394152	2.048597	1.108781
C	0.591166	4.051999	0.197259

C	0.183950	2.325360	2.350105
H	-1.006455	1.151022	0.983853
C	1.174883	4.332025	1.437782
H	0.747263	4.733443	-0.643868
C	0.971984	3.469958	2.518918
H	0.020175	1.644981	3.190052
H	1.788934	5.228324	1.559545
H	1.425947	3.686966	3.489235
C	-1.168655	0.203146	-2.005517
O	-0.720649	-1.024234	-2.376544
C	-0.363308	1.272720	-1.902428
N	-2.536717	0.274972	-1.708826
H	-3.103461	-0.569051	-1.752058
C	-2.353834	2.626259	-1.289885
C	-3.105279	1.477839	-1.426152
C	-3.038767	3.868260	-1.059017
O	-2.498678	4.973862	-0.950174
N	-4.474565	1.500478	-1.299672
H	-5.011659	0.640244	-1.398935
N	-4.435324	3.780276	-0.949775
C	-5.200170	2.646154	-1.049660
O	-6.419272	2.638849	-0.936330
H	-4.938064	4.649459	-0.776759
H	0.682794	1.157986	-2.189664
H	-1.391981	-1.494629	-2.898805

MS16B1

C	-0.916371	2.836016	-1.331177
H	-0.664354	3.750188	-1.888144
C	-0.270165	2.974821	0.049535

C	-0.851633	2.450821	1.212976
C	0.971825	3.623084	0.156325
C	-0.206612	2.566268	2.449604
H	-1.823027	1.953561	1.160963
C	1.617427	3.741075	1.389056
H	1.435486	4.044345	-0.740552
C	1.029487	3.210729	2.542649
H	-0.677415	2.152276	3.345073
H	2.580832	4.253904	1.450599
H	1.530779	3.304462	3.509264
C	-0.941848	0.307912	-1.807947
O	-0.337189	-0.750601	-1.861668
C	-0.334719	1.650669	-2.133720
N	-2.291698	0.328059	-1.506483
H	-2.755088	-0.574911	-1.403387
C	-2.419485	2.719712	-1.295384
C	-3.019832	1.489275	-1.359956
C	-3.246172	3.884814	-1.103236
O	-2.832823	5.041158	-1.002264
N	-4.381414	1.346629	-1.272925
H	-4.810608	0.424217	-1.335717
N	-4.621931	3.632811	-1.026849
C	-5.244946	2.410425	-1.095747
O	-6.455609	2.263267	-1.011540
H	-5.231316	4.438814	-0.891456
H	0.753443	1.569203	-2.018238
H	-0.541916	1.808735	-3.207952

CO_2

C	-6.786928	1.142419	-0.000000

O	-7.349802	2.159602	0.000000
O	-6.224049	0.125239	0.000000
